# Development and validation of an expanded antibody toolset that captures alpha-synuclein pathological diversity in Lewy body diseases

**DOI:** 10.1038/s41531-023-00604-y

**Published:** 2023-12-07

**Authors:** Melek Firat Altay, Senthil T. Kumar, Johannes Burtscher, Somanath Jagannath, Catherine Strand, Yasuo Miki, Laura Parkkinen, Janice L. Holton, Hilal A. Lashuel

**Affiliations:** 1 Laboratory of Molecular and Chemical Biology of Neurodegeneration, Brain Mind Institute, EPFL, Lausanne, Switzerland; 2 Department of Human Genetics, Inselspital, Bern University Hospital, University of Bern, Bern, Switzerland; 3 Queen Square Brain Bank for Neurological Disorders, University College London Queen Square Institute of Neurology, London, England; 4 Department of Neuropathology, Institute of Brain Science, Hirosaki University Graduate School of Medicine, Hirosaki, 036-8562 Japan; 5 Oxford Parkinson’s Disease Centre, University of Oxford, Oxford, UK; 6 Nuffield Department of Clinical Neurosciences, University of Oxford, Oxford, UK

**Keywords:** Neuroscience, Diseases of the nervous system

## Abstract

The abnormal aggregation and accumulation of alpha-synuclein (aSyn) in the brain is a defining hallmark of synucleinopathies. Various aSyn conformations and post-translationally modified forms accumulate in pathological inclusions and vary in abundance among these disorders. Relying on antibodies that have not been assessed for their ability to detect the diverse forms of aSyn may lead to inaccurate estimations of aSyn pathology in human brains or disease models. To address this challenge, we developed and characterized an expanded antibody panel that targets different sequences and post-translational modifications along the length of aSyn, and that recognizes all monomeric, oligomeric, and fibrillar aSyn conformations. Next, we profiled aSyn pathology across sporadic and familial Lewy body diseases (LBDs) and reveal heterogeneous forms of aSyn pathology, rich in Serine 129 phosphorylation, Tyrosine 39 nitration and N- and C-terminal tyrosine phosphorylations, scattered both to neurons and glia. In addition, we show that aSyn can become hyperphosphorylated during processes of aggregation and inclusion maturation in neuronal and animal models of aSyn seeding and spreading. The validation pipeline we describe for these antibodies paves the way for systematic investigations into aSyn pathological diversity in the human brain, peripheral tissues, as well as in cellular and animal models of synucleinopathies.

## Introduction

Synucleinopathies, including Parkinson’s disease (PD), dementia with Lewy bodies (DLB), and multiple system atrophy (MSA), are neurodegenerative diseases characterized by the aggregation and accumulation of the pre-synaptic protein alpha-synuclein (aSyn)^1–3^. Pathological inclusions such as Lewy bodies (LBs), Lewy neurites (LNs), glial cytoplasmic inclusions (GCIs), and neuronal cytoplasmic inclusions (NCIs) are enriched with fibrillar forms of aSyn in PD, DLB, and MSA^1–3^. Familial PD has been linked to variations in the *SNCA* gene that encodes aSyn^4–16^. Furthermore, inoculation of recombinant aSyn fibrils or aSyn aggregates derived from PD and MSA brains can induce LB-like inclusion formation in disease models^17–23^, as well as the spreading of pathology along the gut-brain axis and across brain regions^24–28^.

**Fig. 1:**
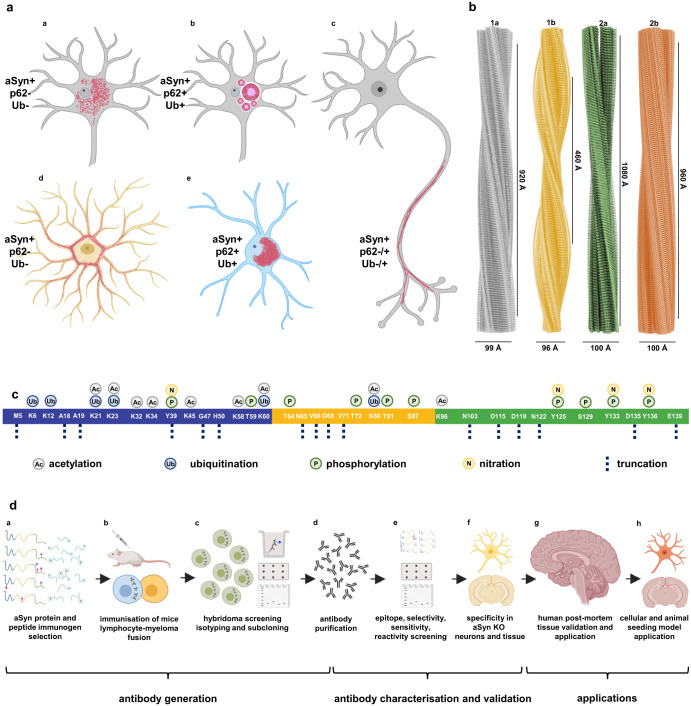
Comprehensive exploration of aSyn heterogeneity - pathology diversity, fibril polymorphism, aSyn PTMs in synucleinopathy brains and antibody generation/ validation steps. **a** Diversity of aSyn pathology in synucleinopathies with **aa** granular/punctate cytoplasmic inclusions in the neurons; **ab** classical LBs in the neuronal soma; **ac** LNs in the neuronal processes; **ad** astrocytic aSyn accumulations; **ae** oligodendroglial cytoplasmic inclusions. These pathological structures show differences in their positivity to aggregation markers, including ubiquitin (Ub) and p62. Schematic created with BioRender.com (agreement no: *QW23G6FJ76*). **b** Cryo-EM three-dimensional reconstructions of the recombinant full-length aSyn PFFs to show the polymorphism of aSyn fibrils generated in vitro^38,39^. Four distinct polymorphs were identified based on the protofilament fold and inter-protofilament interfaces: Polymorph 1a ‘rod’ (PDB-6CU7, EMD-7618); polymorph 1b ‘twister’ (PDB-6CU8, EMD-7619); polymorph 2a (PDB-6SSX, EMD-10307); and polymorph 2b (PDB-6SST, EMD-10305). **c** aSyn PTMs identified in synucleinopathy brain tissues, which include acetylation^29,30,81,82^, ubiquitination^29,30,32,52,72–78^, phosphorylation^7,29,30,50–71^, nitration^79,80^, and truncation^1,29,49,74,76,78,81–96^ across the whole sequence of the protein. **d** A schematic representation of the steps followed for the generation, characterization, validation and application of aSyn antibodies. These involved **da** antibody design via the selection of immunogens comprising of aSyn recombinant proteins and peptides; **db** immunization of the mice followed by lymphocyte–myeloma fusion; **dc** screening of the hybridomas via ELISA, DB and WB, isotyping and subcloning, and **dd** acquisition of purified antibodies. The antibodies were then **de** characterized using a library of aSyn recombinant proteins for their epitopes, conformational selectivity, sensitivity, specificity and reactivity via DB and WB. The antibody specificity was further validated on **df** aSyn KO mouse primary hippocampal and cortical neurons, and in aSyn KO mouse tissue of amygdala. **dg** The antibodies were validated on human brain tissues of different LB diseases. **dh** The mouse aSyn-reactive antibodies were applied to neuronal seeding model and PFF-injected mouse brain tissues to profile the newly formed aggregates. Schematic created with BioRender.com (agreement no: *FN23G6E1SR*). aSyn alpha-synuclein, DB dot/slot blot, cryo-EM cryogenic electron microscopy, ELISA enzyme-linked immunoassay, KO knockout, LB Lewy body, LN Lewy neurite, PFFs pre-formed fibrils, PTM post-translational modification, Ub ubiquitin, WB Western blot.

Diverse forms of aSyn accumulate in brain inclusions associated with synucleinopathies^29–31^, exhibiting variations in morphology^32^, biochemical composition^33–35^, structure^31,36^, and distribution^37^. This heterogeneity is influenced by factors such as synucleinopathy type, cell type, brain region, and patient clinical history (Fig. 1a). Full-length, truncated, and modified recombinant and semi-synthetic aSyn monomers can form fibrils with different conformations in vitro^38,39^ (Fig. 1b). Similarly, fibrils isolated from PD, DLB, and MSA brains exhibit polymorphisms^30,40^.

The study of aSyn has employed antibodies that target sequences, modifications or conformations of the protein to detect and quantify aSyn pathology in LBs, LNs, GCIs and NCIs^41–49^. aSyn accumulating within these inclusions undergo different post-translational modifications (PTMs) at multiple residues (Fig. 1c). These PTMs include phosphorylation^7,29,30,50–71^, ubiquitination^29,30,32,52,72–78^, nitration^79,80^, acetylation^29,30,81,82^ and N- and C-terminal truncations^1,29,49,74,76,78,81–96^. Among these modifications, phosphorylation at Serine 129 (aSyn pS129) is the most common PTM in pathological aSyn aggregates. Increased levels of aSyn pS129 have been observed in PD, DLB, and MSA brains^29,50^. Consequently, aSyn pS129 has emerged as a marker of aSyn pathology. Several antibodies have been developed to target aSyn pS129 and assess the formation and spread of aSyn pathology in the central nervous system and periphery. However, the comprehensive coverage of aSyn pathology by these antibodies has not been thoroughly investigated.

C-terminally cleaved aSyn forms (1–115, 1–119, 1–122, 1–133, 1–135)^1,29,76,78,81–83,87,92,97,98^ and C-terminally nitrated or phosphorylated aSyn at tyrosine residues (Y125, Y133, Y136)^68,69,79,99^, have been identified in LBs and GCIs (Fig. 1c). Truncated species (aSyn 1–119, 1–122) lacking the epitope for aSyn pS129 antibodies are particularly abundant^29,81,82,86,95^. Studies primarily relying on antibodies targeting the C-terminal region, including the PD staging work^100–102^, may potentially miss truncated forms, non-phosphorylated aggregates and aSyn with multiple C-terminal modifications^7,29,68,69,79,103,104^. The cellular environment influences the characteristics of aSyn aggregates^105^, which may have distinct biochemical and structural signatures exposing different amino acid sequences. Therefore, multiple well-characterized antibodies are crucial for understanding disease-specific aSyn features and developing biomarkers for synucleinopathies^1,41,42,45,96^, enhancing our knowledge of disease progression^1,41,42,45,96^.

We aimed to develop an antibody toolbox to detect a range of soluble, aggregated, and post-translationally modified forms of aSyn. We generated novel monoclonal antibodies, complemented these with a pre-existing antibody selection, and systematically assessed their epitope specificity, sensitivity to neighboring PTMs, and conformational selectivity for aSyn using recombinant proteins (Fig. 1d). The antibodies were validated using aSyn knockout (KO) mouse neurons and brain tissues and applied on postmortem synucleinopathy tissues. We selected an antibody subset that covered different regions and post-translationally modified forms of aSyn to profile pathological lesions in LBDs. Our approach revealed distinct and heterogeneously modified aSyn pathologies rich in pS129, Tyrosine 39 (Y39) nitration and N- and C-terminal tyrosine phosphorylations in neurons and glia. This study is the first to use antibodies targeting key disease-associated PTMs to assess aSyn brain pathology in the same set of LBD cases. Additionally, we examined the distribution of differentially modified aSyn aggregates in cellular and animal models, observing hyperphosphorylation during aggregation and inclusion maturation. These findings emphasize the importance of comprehensive toolsets to uncover the diversity of aSyn species in human tissues and model organisms, advancing our understanding of aSyn pathology, neurodegeneration, and disease progression.

## Results

### Design, development, and generation of aSyn monoclonal antibodies

To develop antibodies that capture the diverse biochemical and structural variations of aSyn, we employed a range of human aSyn proteoforms as antigens. These antigens included large peptide fragments and proteins that covered different regions of aSyn, such as the N-terminal, non-amyloid component (NAC), and C-terminal regions, as well as those containing post-translational modifications associated with pathology (Supplementary Table 1). To ensure coverage of disease-associated C-terminal truncations, we incorporated antigen sequences spanning aSyn residues 108–120, 113–127, or 108–140. To increase the chances of generating antibodies targeting the N-terminal and/or NAC region of the protein, we used aSyn 1–20 peptide, as well as human aSyn full-length (1–140) and aSyn 1–119 recombinant proteins, as immunogens. For the generation of aSyn pS129 antibodies, we initiated two programs—one with human aSyn 124–135 peptide phosphorylated at S129 (aSyn 124–135 pS129), and another with human aSyn-120–135 peptide doubly phosphorylated at Y125 and S129 (aSyn-120–135 pY125/pS129). Our goal was to develop phospho-specific antibodies that would detect aSyn pS129 even in the presence of neighboring PTMs. Lastly, to produce monoclonal antibodies against this N-terminal PTM, the mice were immunized with aSyn 34–45 peptide nitrated at Y39 (aSyn 34–45 nY39). Following the immunization of the Bagg’s ALBino color (BALB/c) mice, test bleeds, hybridoma supernatants, and subclones were analyzed via enzyme-linked immunosorbent assay (ELISA), dot/slot blot (DB) and Western blot (WB) against a selected library of aSyn proteins (Supplementary Table 2) to determine the strongest and the most specific antibody response. Details of the monoclonal antibody generation process are provided in the Materials and Methods section. A total of 12 aSyn mouse monoclonal antibodies were obtained (Supplementary Table 3), and their purity was validated by sodium dodecyl sulfate polyacrylamide gel electrophoresis (SDS-PAGE)/Coomassie staining and WB.

### Antibody characterization using recombinant synuclein proteins and peptides

**Fig. 2:**
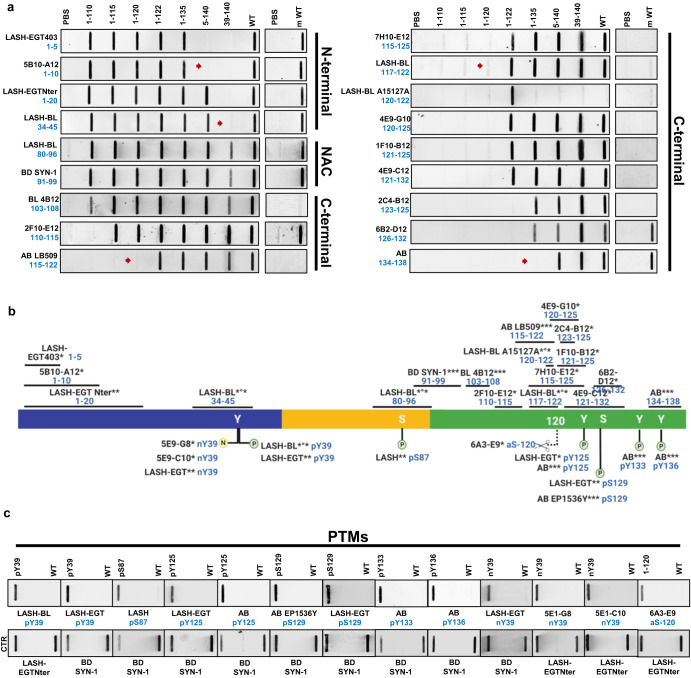
Validation and epitope mapping of aSyn antibodies. **a** DB validation of the novel monoclonal, in-house polyclonal and commercially available aSyn antibodies against the N-terminal, NAC and the C-terminal regions of aSyn for epitope mapping, specificity and species reactivity using a selected library of aSyn recombinant proteins under native conditions. Protein loading control was run via Ponceau S staining. All loaded proteins represent human aSyn forms except for mouse aSyn wild-type (m WT) protein. Red arrows highlight the sensitivities of the antibodies to the presence of neighboring aSyn PTMs. **b** A schematic to represent the novel monoclonal (marked with *), in-house polyclonal (marked with **) and pre-existing commercial aSyn antibodies (marked with ***) included in this study. The commercial antibodies developed jointly with Biolegend are marked with *°*. The epitope information of each antibody is indicated in blue. Schematic created with BioRender.com (agreement no: *JR23G6G5LA*). **c** Specificity validation of the aSyn PTM antibodies via DB screening. aSyn alpha-synuclein, CTR control, DB dot/slot blot, FL full-length, m mouse, NAC non-amyloid component, PBS phosphate-buffered saline, PTM post-translational modification, WT wild-type.

To determine the sequence specificity and epitopes of our antibodies (Fig. 2a), we used a library of aSyn recombinant protein standards (Supplementary Table 2). The two antibodies, LASH-EGT403 and 5B10-A12, targeted the N-terminus of aSyn residues 1–5 and 1–10, respectively. All the other novel monoclonal antibodies were mapped to the C-terminal region spanning residues 110–132 and showed staggered coverage of this protein region (Fig. 2a, b and Supplementary Table 3). The epitopes of these seven antibodies were mapped to residues 110–115 (2F10-E12), 115–125 (7H10-E12), 120–125 (4E9-G10), 121–125 (1F10-B12), 121–132 (4E9-C12), 123–125 (2C4-B12), and 126–132 (6B2-D12). The results of the epitope mapping of all the novel monoclonal antibodies are summarized in Fig. 2b (antibodies marked with *).

The newly produced monoclonal antibodies alone were not sufficient to cover all regions of the aSyn sequence. Therefore, we incorporated in-house polyclonal aSyn antibodies (Fig. 2b, antibodies marked with **), as well as commercially available and frequently used aSyn antibodies (Fig. 2b, antibodies marked with ***) within the study. Of these commercial antibodies, LASH-BL 34–45, LASH-BL 80–96, LASH-BL 117–122, LASH-BL A15127A (120–122), and LASH-BL pY39 were developed jointly with Biolegend (Fig. 2b, antibodies marked with *°*). The epitopes and sequence specificities of all these antibodies were validated by DB (Fig. 2a). We mapped the epitope of the homemade polyclonal LASH-EGTNter antibody to aSyn residues 1–20. LASH-BL A15127A showed a strong preferential positivity to aSyn truncated at residue 122. However, further validation is needed to confirm if this antibody is specific to truncated aSyn (1–122; Fig. 2a). For all the other commercial antibodies, the epitopes we identified were similar to those reported in the literature (Fig. 2a, b).

In addition to the antibodies against non-modified aSyn, we also generated novel monoclonal antibodies that target specific aSyn PTMs. Three aSyn PTM antibodies were developed, two of which were mapped to aSyn nY39 (5E1-G8 and 5E1-C10), and one to aSyn truncated at residue 120 (6A3-E9) by DB and WB (Fig. 2b, c and Supplementary Fig. 1a). Among the monoclonal antibodies against aSyn nY39, 5E1-G8 was weakly positive to aSyn WT and aSyn nY39 by WB (Supplementary Fig. 1a, red arrow) but not by DB (Fig. 2c). On the other hand, 5E1-C10 was positive only for aSyn nY39 both by DB and WB (Fig. 2c and Supplementary Fig. 1a). The specificity of 6A3-E9 (aSyn-120) was further validated via supplementary DB, WB and surface plasmon resonance (SPR) analyses (Supplementary Fig. 1b–d), all of which showed that it detects human aSyn cleaved at residue 120, but not the full-length protein. We complemented our battery of antibodies against the aSyn PTMs with the previously generated in-house polyclonal antibodies against aSyn nY39 (LASH-EGT nY39), aSyn pY39 (LASH-EGT pY39), aSyn pS87 (LASH pS87), aSyn pY125 (LASH-EGT pY125), aSyn pS129 (LASH-EGT pS129), the monoclonal aSyn pY39 antibody generated in collaboration with Biolegend (LASH-BL pY39) and the commercially available aSyn pY125 (AB pY125), pS129 (AB EP1536Y), pY133 (AB pY133), and pY136 (AB pY136) antibodies (Fig. 2b). These were validated and found to be specific to their targeted modifications by DB (Fig. 2b, c). Altogether, this selection process provided us with 18 aSyn non-modified and 13 aSyn PTM antibodies to work with. All antibodies used in this study are listed in Supplementary Table 4.

We next assessed the ability of the antibodies to recognize human and mouse aSyn. All N-terminal and NAC region antibodies, including LASH-EGT403 (1–5), 5B10-A12 (1–10), LASH-EGTNter (1–20), LASH-BL (34–45), LASH-BL (80–96) and BD SYN-1 (91–99) recognized both human and mouse aSyn (Fig. 2a). The large majority of the C-terminal antibodies, on the other hand, detected only human aSyn proteins, with the exception of 2F10-E12 (110–115), 6B2-D12 (126–132) and Abcam (134–138) antibodies that detected human and mouse aSyn (Fig. 2a). These findings are consistent with the sequence differences between human and mouse aSyn in the C-terminus, particularly in the region covering residues 115–125 (Supplementary Fig. 2a).

We have recently shown that the aSyn pS129 antibody binding could be affected by the presence of aSyn PTMs neighboring S129^106^. Therefore, we investigated the sensitivity of the non-modified aSyn antibodies to the presence of PTMs in close proximity to their epitopes by DB, and the results are summarized in Table 1. A large number of the aSyn C-terminal antibodies (i.e., 8 out of 12) showed sensitivity to the presence of aSyn pY125 and/or truncations within residues 120–125 (Table 1). When aSyn pY125 was present, for instance, antibodies 7H10-E12 (115–125), 4E9-G10 (120–125), 4E9-C12 (121–132) and 2C4-B12 (123–125) failed to produce a strong positive result by DB (Supplementary Fig. 2c, red arrows). This finding is in line with the mapped epitopes of these antibodies (Fig. 2b), which cover Y125. Therefore, phosphorylation of this residue may interfere with antibody recognition of aSyn. Similarly, the C-terminal 6B2-D12 (126–132) antibody did not detect aSyn pS129 protein (Supplementary Fig. 2c, red arrow). The positive signal revealed by AB LB509 (115–122), and LASH-BL 117–122 were weaker when aSyn was truncated at residue 120 (Fig. 2b, red arrows) or when C-terminal residues 120–125 were absent (Supplementary Fig. 2c, red arrows). AB 134–138 antibody did not show any positive signal when aSyn was truncated at residue 135 (Fig. 2b, red arrow) but was not affected by the presence of phosphorylation at Y136 (Supplementary Fig. 2c). We expanded the same analysis to the N-terminal and NAC region antibodies (Table 1). The N-terminal 5B10-A12 (1–10) showed no positivity when aSyn was truncated at residue 5 (Fig. 2a, red arrow), which is consistent with the epitope identification of this antibody. The LASH-BL 34–45 antibody showed no sensitivity to the presence of nY39 (Supplementary Fig. 2c) but failed to detect aSyn when it was truncated at residue 39 (Fig. 2a, red arrow). Detection of aSyn by the other antibodies was not affected by the presence of neighboring PTMs (Table 2). These results highlight that the antibody sensitivities to the presence of PTMs deserve consideration before aSyn antibodies are selected for prospective experimental studies.

**Table 1:** Characterization of non-modified aSyn antibodies using recombinant synuclein proteins and peptides.

Antibody	Epitope	Species reactivity	bSyn detection	gSyn detection	Other amyloidogenic protein reactivity	Neighboring PTM sensitivity	Conformational selectivity	
							WB	DB
LASH-EGT403	1–5	hu, mus	+	+	–	–	m/o/f	m/o/f
5B10-A12	1–10	hu, mus	–	–	–	M5	m/o/f	m/o/f
LASH-EGTNter	1–20	hu, mus	+	–	–	–	m/o/f	m/o/f
LASH-BL 34–45	34–45	hu, mus	+	–	–	Y39	m/f	m/o/f
LASH-BL 80–96	80–96	hu, mus	–	–	–	–	m/o/f	m/o/f
BD SYN-1	91–99	hu, mus	–	–	–	–	m/o/f	m/o/f
BL 4B12	103–108	hu	–	–	–	–	m/o/f	m/o/f
2F10-E12	110–115	hu, mus	–	–	–	–	m/o/f	m/o/f
AB LB509	115–122	hu	–	–	–	P120-Y125	m/o/f	m/o/f
7H10-E12	115–125	hu	–	–	–	pY125	m/o/f	m/o/f
LASH-BL 117–122	117–122	hu	–	–	–	P120-Y125	m/o/f	m/o/f
LASH-BL A15127A	120–122	hu	–	–	–	–	m/o/f	m/o/f
4E9-G10	120–125	hu	–	–	–	pY125	m/o/f	m/o/f
1F10-B12	121–125	hu	–	–	–	–	m/o/f	m/o/f
4E9-C12	121–132	hu	–	–	–	pY125	m/o/f	m/o/f
2C4-B12	123–125	hu	–	–	–	pY125	m/o/f	m/o/f
6B2-D12	126–132	hu, mus	–	–	+/TDP-43	pS129	m/o/f	m/o/f
AB 134–138	134–138	hu, mus	+	–	–	D135	m/o/f	m/o/f

= truncation, *aSyn* alpha-synuclein, *bSyn* beta-synuclein, *DB* dot blot, *f* fibril, *gSyn* gamma-synuclein, *hu* human, *m* monomer, *mus* mouse, *o* oligomer, *PTM* post-translational modification, *TDP-43* transactive response DNA-binding protein 43 kDa, *WB* Western blot.

**Table 2:** Specificity summary of aSyn antibodies on aSyn KO mouse neuronal culture and brain tissues.

Antibody	Epitope	Specificity: aSyn KO neurons - ICC	Specificity: aSyn KO neurons—WB		Specificity: aSyn KO tissue—IHC
			15 kDa	35-180 kDa	
LASH-EGT403	1–5	+	+	–	+
5B10-A12	1–10	+	+	–	+
LASH-EGTNter	1–20	+/bSyn-positive	+	–	+
LASH-BL 34–45	34–45	+/bSyn-positive	+	–	+
LASH-BL 80–96	80–96	−/hipp aSyn KO only	+	–	+
BD SYN-1	91–99	+	+	+	+
BL 4B12	103–108	−/mild nuclear	+	–	+
2F10-E12	110–115	+	+	–	+
AB LB509	115–122	+	+	–	+
7H10-E12	115–125	–	+	+	+
LASH-BL 117–122	117–122	–	+	+	+
LASH-BL A15127A	120–122	–	+	–	+
4E9-G10	120–125	+	+	–	+
1F10-B12	121–125	–	+	+	+
4E9-C12	121–132	+	+	+	+
2C4-B12	123–125	–	+	+	+
6B2-D12	126–132	+	+	–	+
AB 134–138	134–138	+/bSyn-positive	+/bSyn-positive	–	+
LASH-BL pY39	pY39	+	+	–	+
LASH-EGT pY39	pY39	+	+	–	+
LASH pS87	pS87	−/mild nuclear	+	+	−/punctate cytoplasmic
LASH-EGT pY125	pY125	+	+	+	−/punctate cytoplasmic
AB pY125	pY125	+	+	+	+
AB EP1536Y	pS129	+	+	+	+
LASH-EGT pS129	pS129	+	–	–	−/punctate cytoplasmic
AB pY133	pY133	+	+	+	+
AB pY136	pY136	+	+	+	+
LASH-EGT nY39	nY39	+	+	+	+
5E1-G8	nY39	–	+	–	−/mild diffuse background
5E1-C10	nY39	–	+	–	−/mild diffuse background
6A3-E9	aSyn-120	+	+	+	+

*+* specific, *−* non-specific, *aSyn* alpha-synuclein, *bSyn* beta-synuclein, *hipp* hippocampus, *ICC* immunocytochemistry, *IHC* immunohistochemistry, *kDa* kilodalton, *KO* knockout, *WB* Western blot.

The synuclein family of proteins consists of aSyn, beta-synuclein (bSyn) and gamma-synuclein (gSyn; Supplementary Fig. 2b)^107^, which share significant sequence homology. We investigated the reactivity of the antibodies targeting non-modified aSyn to bSyn and gSyn. We note that the N-terminal LASH-EGT403 (1–5) was the only antibody to detect aSyn, bSyn and gSyn, therefore acting as a pan-synuclein antibody (Supplementary Fig. 2d, blue and green arrows). This is consistent with the fact that the first four residues of all the synuclein proteins are identical (Supplementary Fig. 2b). The LASH-EGTNter (1–20), LASH-BL (34–45) and the extreme C-terminal antibody AB (134–138) showed reactivity also to bSyn (Supplementary Fig. 2d, blue arrows) but not to gSyn. The N-terminal antibody 5B10-A12 (1–10), the NAC region antibodies and the rest of the C-terminal antibodies did not recognize bSyn or gSyn (Supplementary Fig. 2d). These results are in line with the sequence similarities and differences between aSyn, bSyn and gSyn (Supplementary Fig. 2b).

We next assessed the specificity of the antibodies against other amyloidogenic proteins including tubulin-associated unit (tau), amyloid-beta (a-beta), and transactive response DNA-binding protein 43 kDa (TDP-43). None of the aSyn antibodies revealed any positivity to these amyloidogenic proteins (Supplementary Fig. 2d), except for the C-terminal 6B2-D12 (126–132), which was positive for recombinant TDP-43 by DB (Supplementary Fig. 2d, red arrow). These results confirmed that 17 out of 18 non-modified aSyn antibodies are specific for recombinant synuclein and do not recognize other amyloidogenic recombinant proteins.

**Fig. 3:**
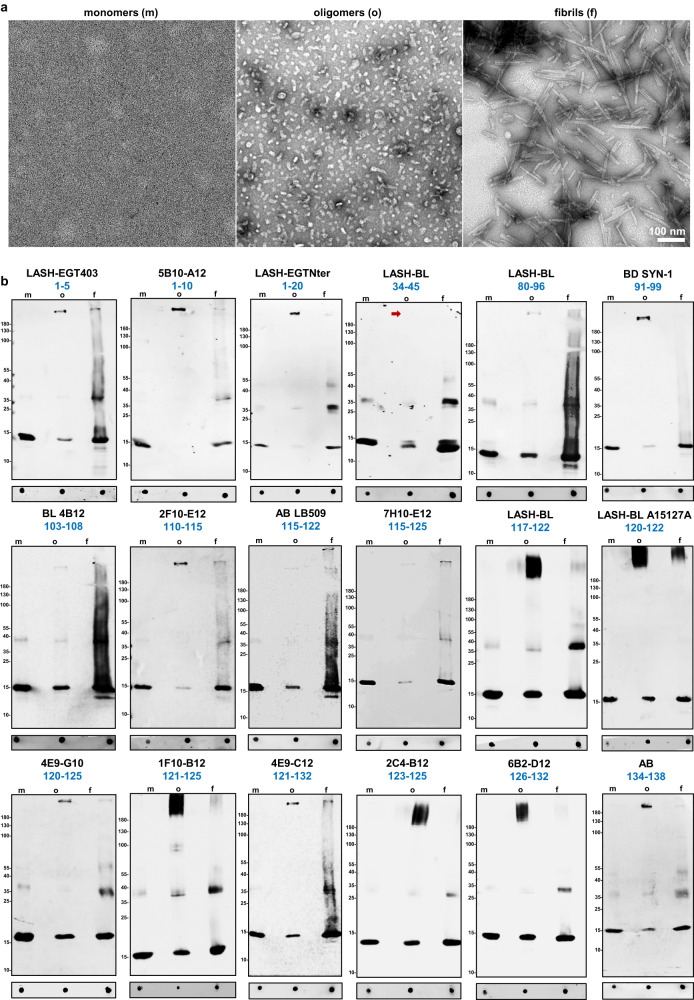
Selectivity of aSyn antibodies over aSyn conformations. **a** Representative EM images of aSyn human WT monomers, oligomers and fibrils. **b** DB and WB characterization of 18 aSyn antibodies to determine their conformational selectivity using aSyn human WT recombinant monomers, oligomers and fibrils. aSyn alpha-synuclein, DB dot/slot blot, EM electron microscopy, f fibrils, m monomers, o oligomers, WB Western blot, WT wild-type.

aSyn exists in different conformations in healthy and diseased human tissues^2,3,41,47,108,109^. To determine the selectivity of the antibodies towards the different aSyn conformations, we used DB and WB to assess their ability to recognize purified recombinant aSyn WT monomers, oligomers, and fibrils (Fig. 3a, b and Table 1), which were prepared and characterized as previously described^110^. Electron microscopy (EM) analysis confirmed the aggregation state of aSyn in all three preparations (Fig. 3a). All antibodies recognized the three forms of aSyn by DB but showed differential ability to detect SDS-resistant high molecular weight (HMW) oligomers by WB. Namely, LASH-BL 34–45, which detected aSyn monomers, oligomers, and fibrils by DB, did not recognize HMW aSyn oligomers under denatured conditions (Fig. 3b, red arrow). These results suggest that the vast majority of the selected antibodies are capable of recognizing different conformations and aggregation states of aSyn.

### Antibody validation using aSyn KO mouse primary neurons and brain tissues

To validate the specificity of the antibodies to aSyn species in cells, we assessed their immunoreactivity using primary neurons derived from C57BL/6J-OlaHsd mice (*SNCA* − /−) lacking expression of mouse aSyn. We first determined the minimal working concentration for each of the mouse-reactive antibodies on WT naïve and mouse aSyn pre-formed fibril(PFF)-seeded hippocampal neurons by immunocytochemistry (ICC). This approach allowed us to work on aSyn KO neurons with appropriate antibody concentrations that otherwise permit the detection of endogenous aSyn, exogenously added aSyn fibrils, and the newly formed aSyn aggregates in WT neurons, based on a well-characterized neuronal seeding model^20,21,23^. For the antibodies that are human-reactive only, we opted for the recommended dilutions by the suppliers when such information was available, and if not, we aimed at a final antibody concentration of 2–5 µg/mL for ICC. We then screened the antibodies using aSyn KO hippocampal and cortical neurons.

By ICC, the N-terminal LASH-EGT403 (1–5), 5B10-A12 (1–10), the NAC region BD SYN-1 (91–99), the C-terminal 2F10-E12 (110–115), AB LB509 (115–122), 4E9-G10 (120–125), 4E9-C12 (121–132) and 6B2-D12 (126–132) antibodies showed no non-specific background in hippocampal (Supplementary Fig. 3a) and cortical (Supplementary Fig. 3b) aSyn KO neurons. The LASH-EGTNter (1–20), LASH-BL (34–45) and the C-terminal AB (134–138) antibodies were positive, possibly for bSyn protein, both in hippocampal and cortical aSyn KO neurons (Supplementary Fig. 3a, b, blue arrows), an observation consistent with DB results on recombinant bSyn reactivity using these antibodies (Table 1 and Supplementary Fig. 2d, blue arrows). Collectively, all of these antibodies showed specificity by ICC on aSyn KO hippocampal and cortical neurons. The cytoplasmic and/or nuclear signals detected in hippocampal and cortical aSyn KO neurons with the C-terminal BL 4B12 (103–108), 7H10-E12 (115–125), LASH-BL (117–122), LASH-BL A15127A (120–122), 1F10-B12 (121–125) and 2C4-B12 (123–125), on the other hand, were deemed to be non-specific (Supplementary Fig. 3a, b, red arrows). Interestingly, the NAC region LASH-BL 80–96 antibody revealed a weak, non-specific cytoplasmic signal in the hippocampal (Supplementary Fig. 3a, red arrow) but not in cortical aSyn KO neurons (Supplementary Fig. 3b).

Next, we assessed the specificity of the aSyn PTM antibodies in aSyn KO neurons by ICC. The LASH-BL pY39, LASH-EGT pY39, LASH-EGT pY125, AB pY125, AB EP1536Y pS129, LASH-EGT pS129, AB pY133, AB pY136, LASH-EGT nY39, and 6A3-E9 antibodies were negative by ICC (Supplementary Fig. 4a, b), suggesting that these antibodies are specific and show no cross-reactivity. In contrast, the monoclonal aSyn nY39 antibodies 5E1-G8 and 5E1-C10 were strongly positive both in the hippocampal and cortical aSyn KO neurons, showing non-specific background. Mild nuclear positivity was observed with LASH pS87 (Supplementary Fig. 4a, b, red arrows).

We used a similar approach to investigate the specificity of the aSyn antibodies in neuronal lysates by WB. Sequential extraction was run on aSyn KO hippocampal and cortical neurons, and the soluble and insoluble fractions were profiled using the aSyn antibodies. aSyn mouse or human recombinant standards were used as positive controls. Of the human-reactive non-modified aSyn antibodies, faint non-specific bands were present mainly in the HMW regions of the soluble and/or insoluble fractions of the C-terminal BL 4B12 (103–108), AB LB509 (115–122), LASH-BL A15127A (120–122), 4E9-G10 (120–125) (Supplementary Fig. 5a, red arrows), but not in the region where monomeric aSyn migrates, at 15 kDa. Non-specific bands were not observed with the human-reactive 7H10-E12 (115–125), LASH-BL (117–122), 1F10-B12 (121–125), 4E9-C12 (121–132) and 2C4-B12 (123–125) antibodies (Supplementary Fig. 5a). The human- and mouse-reactive non-modified aSyn antibodies i.e., the N-terminal LASH-EGT403 (1–5), 5B10-A12 (1–10), LASH-EGTNter (1–20), LASH-BL (34–45), the NAC region LASH-BL (80–96), BD SYN-1 (91–99), the C-terminal 2F10-E12 (110–115), 6B2-D12 (126–132) and AB (134–138) antibodies, did not detect any non-specific bands at 15 kDa in the phosphate-buffered saline(PBS)-treated soluble or insoluble fractions (Supplementary Fig. 5a), with AB (134–138) possibly detecting bSyn in the PBS-treated soluble neuronal fractions (Supplementary Fig. 5a, blue arrows). On the other hand, the N-terminal LASH-EGT403, 5B10-A12, LASH-EGTNter, LASH-BL 34–45, the NAC region LASH-BL 80–96, the C-terminal 2F10-E12, 6B2-D12 and AB 134–138 antibodies showed non-specific bands in the HMW regions in the soluble and/or insoluble fractions (Supplementary Fig. 5a, red arrows). Interestingly, most of the aSyn PTM antibodies did not reveal any non-specificity, except for LASH-BL pY39, LASH-EGT pY39, 5E1-G8 nY39 and 5E1-C10 nY39 that detected a few non-specific bands between 35 and 180 kDa, and LASH-EGT pS129 which detected both HMW bands and bands around 15 kDa in the insoluble fractions (Supplementary Fig. 5b, red arrows). We summarized these results in Table 2.

Next, we validated the specificity of the aSyn antibodies on aSyn KO mouse amygdala tissue, a brain region previously shown to be particularly affected by aSyn pathology^111^. By immunofluorescence (IF), there was no background with any of the antibodies against non-modified aSyn (Supplementary Fig. 6a). With the aSyn PTM antibodies, on the other hand, we observed non-specific punctate cytoplasmic positivity with LASH pS87, LASH-EGT pY125, LASH-EGT pS129 and mild diffuse background with 5E1-G8 and 5E9-C10 nY39 antibodies (Supplementary Fig. 6b, red arrows). A summary of the findings on aSyn KO brain tissue for each antibody is presented in Table 2.

### Antibody validation and application in human postmortem brain tissues

**Fig. 4:**
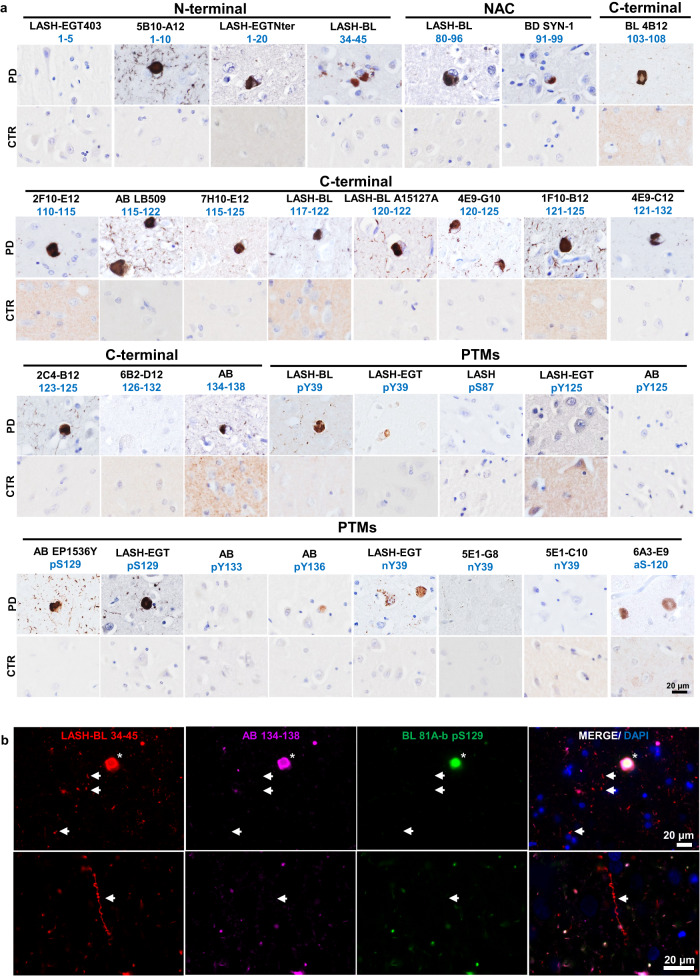
Application and validation of the aSyn antibodies on PD tissue. **a** The in-house monoclonal, polyclonal and commercial aSyn antibodies were optimized for IHC on the PD cingulate cortex. Non-specific staining was not observed in age-matched healthy controls. Representative images taken from the cortical deep grey matter (layers V–VI) of PD1, CTR1 and CTR2. **b** Triple labeling of PD cingulate cortex by IF using an aSyn N-terminal (LASH-BL 34–45), a C-terminal (AB 134–138) and a pS129 (BL 81A-biotin) antibody. LBs are marked with asterisks, and LNs with arrows. Representative images from PD1 cingulate cortex are taken using Leica DM5500 B upright microscope at ×20 magnification. aSyn alpha-synuclein, CTR control, DAPI 4’,6-diamidino-2-phenylindole, IF immunofluorescence, IHC immunohistochemistry, LB Lewy body, LN Lewy neurite, NAC non-amyloid component, PD Parkinson’s disease, PTM post-translational modification.

Following the validation of the antibodies using recombinant proteins, aSyn KO mouse primary neurons, and brain samples, we applied these tools on human postmortem formalin-fixed paraffin-embedded (FFPE) brain tissues. The antibodies were titrated for immunohistochemistry (IHC) by comparing different pre-treatment (i.e., epitope retrieval) conditions and antibody dilutions. All of the non-modified aSyn antibodies (18) revealed moderate to extensive staining of aSyn pathology in the PD cingulate cortex (Fig. 4a) except for the N-terminal LASH-EGT403 (1–5) and C-terminal 6B2-D12 (126–132) antibodies, which were rarely immunoreactive to LBs and did not detect LNs.

Among the three PTM antibodies against aSyn nY39, only the LASH-EGT nY39 polyclonal antibody positively detected cortical LBs and LNs. The 5E1-G8 and 5E1-C10 nY39 monoclonal antibodies, on the other hand, were negative on PD tissue by IHC. With regards to aSyn phosphorylation, some LBs but also thin neurites were positive for aSyn pY39, detected both by the monoclonal LASH-BL pY39 and by the polyclonal LASH-EGT pY39 antibodies. Likewise, extensive LB and LN pathology was revealed both by the commercial AB EP1536Y (pS129) and the homemade LASH-EGT pS129 antibodies. On the contrary, the LASH pS87 and LASH-EGT pY125 antibodies showed little to no reactivity in the PD cingulate cortex. Whilst we could not detect any positivity in this particular PD cingulate cortex with AB pY125 and AB pY133, these antibodies work by IHC to reveal LBs and neuritic pathology in LBD tissues, as shown in our previous study^103^. This suggests that these antibodies specifically detect C-terminal tyrosine phosphorylations on postmortem human tissues when this modification are present. The truncation-specific antibody 6A3-E9 (aSyn-120) detected exclusively the LBs without producing signal in the neurites (Fig. 4a).

Interestingly, triple immunolabelling using aSyn N-terminal, C-terminal, and PTM antibodies revealed that the cortical LBs were equally detected by all antibodies (Fig. 4b and Supplementary Fig. 7, asterisks). Yet, some of the LNs and neuropil dots were selectively revealed by the N-terminal LASH-BL 34–45 antibody, and not by the C-terminal AB 134–138 or the 81 A pS129 antibodies (Fig. 4b and Supplementary Fig. 7 arrows), suggesting that a portion of neuritic pathology is non-phosphorylated and may be cleaved in the extreme C-terminus.

### Panel selection of aSyn antibodies reveals the biochemical and morphological diversity of human aSyn pathology

**Fig. 5:**
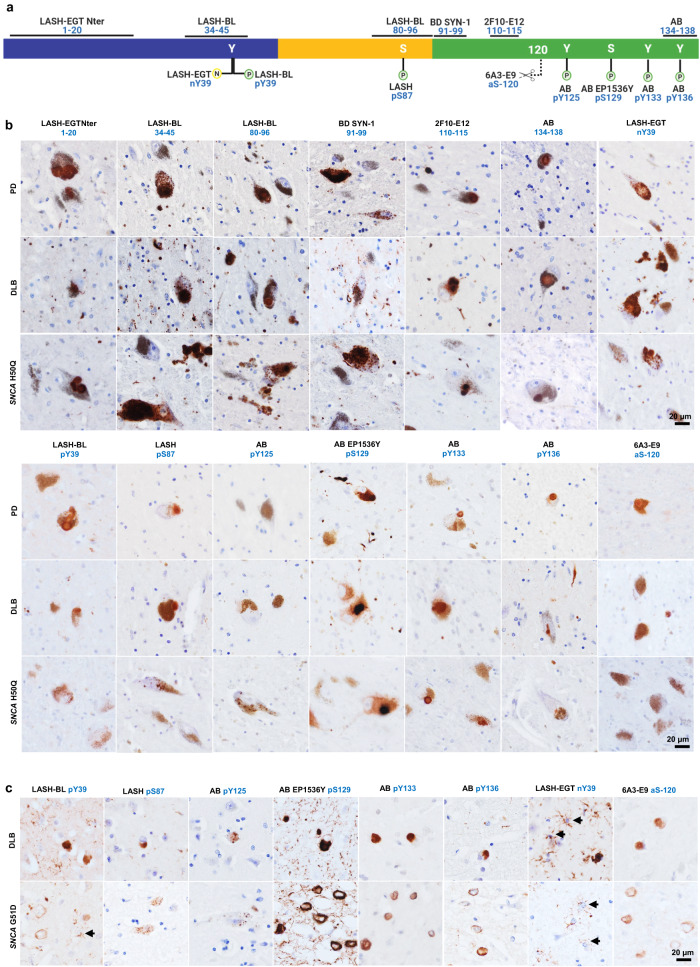
A selected panel of aSyn antibodies reveal the broad diversity of human pathology in the SN of LBDs. **a** An outline to show the epitopes of the aSyn antibody selection used for IHC studies on LBD tissues. Schematic created with BioRender.com (agreement no: *NU23G6E7KK*). **b** Representative images from the substantia nigra of sporadic (PD, DLB) and familial (*SNCA* H50Q) LBDs screened with the selection of aSyn non-modified and PTM antibodies. Images taken from the SN of PD2, PD3, DLB1, and *SNCA* H50Q1. **c** Representative images from the cingulate cortex of sporadic (DLB) and familial (*SNCA* G51D) LBDs screened with the selected aSyn PTM antibodies. Images taken from the cortical deep grey matter (layers V–VI) of DLB1 and *SNCA* G51D1. Arrows indicate star-shaped astroglial aSyn accumulations. aSyn alpha-synuclein, DLB dementia with Lewy bodies, IHC immunohistochemistry, LBD Lewy body disease, SN substantia nigra, PD Parkinson’s disease, PTM post-translational modification.

In human tissues, aSyn displays heterogeneous conformations and proteoforms^29,32,45,50,79,96,99^. To further investigate aSyn pathology in various types of LBDs, we carefully selected a subset of highly specific and effective antibodies that offers extensive coverage of the aSyn sequence and its post-translational modifications. We included two antibodies against the N-terminus (LASH-EGTNter 1–20 and LASH-BL 34–45), two antibodies against the NAC region (LASH-BL 80–96 and BD SYN-1 91–99), and two antibodies against the C-terminus (i.e., 2F10-E12110–115 and AB 134–138) of aSyn. Two antibodies were incorporated to cover the aSyn serine phosphorylations: AB EP1536Y pS129, as this antibody has been the most specific to aSyn pS129 species in our hands and in the literature^106,112^, and LASH pS87, which was recently shown to detect LBs in PD and GCIs in MSA^113^. For the N-terminal tyrosine phosphorylation modification, we opted for the monoclonal LASH-BL pY39 antibody. For the C-terminal phosphorylation at Y125, we selected the polyclonal aSyn pY125 antibody from Abcam as it did not show non-specific background signal in aSyn KO mouse brain tissues (Supplementary Fig. 6b). To cover all possible C-terminal tyrosine phosphorylation modifications, we also included the polyclonal aSyn pY133 and pY136 antibodies from Abcam. To the best of our knowledge, these two C-terminal tyrosine phosphorylations have not been explored in postmortem tissues, with the exception of one recent study^71^. Lastly, we added 6A3-E9 against aSyn 1-120 and LASH-EGT nY39 to our selection. The final antibody subset (14) chosen for further human tissue screening is shown in Fig. 5a.

We validated the specificity of these 14 antibodies to aSyn species directly in human brain tissue using postmortem brain samples with neurodegenerative diseases that are not classified as synucleinopathies. The frontal cortices of progressive supranuclear palsy (PSP) and corticobasal degeneration (CBD), and the hippocampi and entorhinal cortices of Alzheimer’s disease (AD), Pick’s disease (PiD) and frontotemporal lobar degeneration of TDP-43 type C (FTLD-TDP/C) cases were immunostained. None of the non-modified aSyn antibodies showed any immunoreactivity (Supplementary Fig. 8). With regards to the aSyn PTM antibodies, LASH-EGT nY39, LASH-BL pY39, AB pY133, and 6A3-E9aSyn-120 were negative in all cases stained (Supplementary Fig. 8). LASH pS87 and AB pY125 detected sparse granular cytoplasmic aSyn, and AB EP1536Y (pS129) some neuritic aSyn in AD entorhinal cortex (Supplementary Fig. 8, arrows). In addition, LASH pS87 revealed rare cytoplasmic positivity in PiD and AB pY136 in FTLD-TDP/C hippocampi (Supplementary Fig. 8, arrows). Given that aSyn accumulation within the inclusions has been reported in AD^114^ and PiD^115^ postmortem brains, we believe that these positive structures may represent genuine aSyn species.

The selected aSyn antibodies were then used to immunohistochemically analyze the substantia nigra of sporadic (PD *n* = 6, PDD *n* = 2, DLB *n* = 1) and familial (*SNCA* G51D *n* = 3, *SNCA* H50Q *n* = 1, *SNCA* duplication *n* = 1) LBDs, and the results summarized in Table 3. The LBs and LNs were uniformly detected by all aSyn non-modified and PTM antibodies except for AB pY125 and 6A3-E9 (aSyn-120) (Fig. 5b, Supplementary Fig. 9a, and Table 3). The neuronal diffuse cytoplasmic aSyn with or without an aggregate was likewise revealed by all antibodies apart from LASH-BL pY39 and AB pY136 (Table 3). The neuronal punctate cytoplasmic aSyn species were revealed only by a subgroup of the non-modified aSyn antibodies, namely LASH-BL 34–45, LASH-BL 80–96, and BD SYN-1 (91–99) (Fig. 5b and Supplementary Fig. 9a). Similarly, the astroglial accumulations were exclusively revealed by these three non-modified aSyn antibodies and the LASH-BL pY39 and the LASH-EGT nY39 antibodies, and not by any other antibodies included in the study (Table 3). Interestingly, in the cingulate cortex of these LBDs, we observed a similar phenomenon where LASH-BL pY39 and LASH-EGT nY39 antibodies picked up star-shaped glial structures (Fig. 5c, arrows and Supplementary Fig. 9b). The presence of coiled body-like oligodendroglial accumulations of aSyn in the substantia nigra has been reported previously^116^. These oligodendroglial species were revealed best by the C-terminal 2F10-E12 (110–115) antibody (Table 3). The extrasomal aSyn species were detected by all aSyn non-modified and PTM antibodies except for AB pY125 and were particularly enhanced with truncation at residue 120 as well as nitration at Y39 (Fig. 5b, Supplementary Fig. 9a, and Table 3).

**Table 3:** A summary of the pathology detection patterns of aSyn non-modified and PTM antibodies on LBD substantia nigra.

Antibody	Neuronal				Glial		Extrasomal
	LBs	Diffuse cytoplasmic with an aggregate	Punctate cytoplasmic	LNs & neuropil dots	Astroglial	Coiled body-like oligodendroglial	
LASH-EGTNter 1–20	++	++	–	++	–	+	+
LASH-BL 34–45	+++	+	+++	+++	+++	+	+
LASH-BL 80–96	++	+	++	++	++	+	+
BD SYN-1 91–99	++	+	++	++	++	+	+
2F10-E12 110–115	+++	++	–	+++	–	++	+
AB 134–138	++	++	–	++	–	–	+
LASH-BL pY39	+	–	–/+	+	+	+	+
LASH pS87	+	+	–/+	+	–	–	+
AB pY125	–	+	–/+	–	–	–	–
AB EP1536Y pS129	+++	++	–	+++	–	+	+
AB pY133	+	+	–	+	–	–	+
AB pY136	++	–	–	++	–	–	+
LASH-EGT nY39	+	+	++	+	++	–	++
6A3-E9 aSyn-120	–	+	–	–	–	–	++

*−* absent, *+* mild, *++* moderate, *+++* frequent, *aSyn* alpha-synuclein, *LB* Lewy body, *LBD* Lewy body disease, *LN* Lewy neurite, *PTM* post-translational modification.

Both in the substantia nigra and the cingulate cortex, the most abundant aSyn PTM was aSyn pS129, and the AB EP1536Y antibody against this modification labeled LBs, diffuse neuronal cytoplasmic aSyn, neurites and neuropil dots extensively (Fig. 5b, c and Supplementary Fig. 9a, b). In contrast, LASH pS87 together with AB pY125 revealed only sparse structures across the LBDs. LASH pS87 antibody unveiled very rare puncta in the neuronal cytoplasm, dystrophic neurites and diffuse neuronal cytoplasmic accumulations in the substantia nigra (Fig. 5b and Supplementary Fig. 9a) but no glial accumulations (Fig. 5b, Supplementary Fig. 9a, and Table 3). AB pY125 labeled only very rare punctate cytoplasmic aSyn structures and rare diffuse cytoplasmic inclusions in the substantia nigra, without staining any neurites or LBs in either of the two regions examined (Fig. 5b, c and Supplementary Fig. 9a, b). The other C-terminal tyrosine phosphorylation antibody AB pY133 detected some neurons containing multiple LBs, diffuse neuronal cytoplasmic inclusions and rare thin threads, whereas AB pY136 moderately picked up LBs, dystrophic neurites, neuropil dots, and thin threads (Fig. 5b, c and Supplementary Fig. 9a, b). The truncation-specific 6A3-E9 antibody (aSyn-120), on the contrary, stained diffuse neuronal cytoplasmic and extrasomal aSyn structures, but no neurites (Fig. 5b, c and Supplementary Fig. 9a, b). Together, our research revealed a wide spectrum of abnormal aSyn accumulations in both neurons and glial cells within LBD brains (Table 3). We found that specific modifications of aSyn vary depending on the cell type, and we identified various combinations of modified aSyn species that have not been thoroughly documented before. Notably, we discovered the presence of aSyn nY39, pY133, and pY136, which have not been previously described in a comprehensive manner.

### Biochemical and morphological diversity of aSyn aggregates in cellular and animal seeding models

Seeding-based cellular and animal models using aSyn PFFs have emerged as the most common tools to investigate mechanisms and pathways of aSyn pathology formation^17–21,23,117^. To characterize the diversity of aSyn species in these PFF models, we investigated the ability of our antibodies to detect exogenously added PFFs in aSyn KO hippocampal and cortical neurons by ICC and WB (Supplementary Figs. 3–5) 14 h after treatment. Our findings are summarized in Table 4. All of the nine non-modified and mouse aSyn-reactive antibodies i.e., the N-terminal LASH-EGT403 (1–5), 5B10-A12 (1–10), LASH-EGTNter (1–20) and LASH-BL 34–45; the NAC region LASH-BL 80–96, BD SYN-1 (91–99); and the C-terminal 2F10-E12 (110–115), 6B2-D12 (126–132) and AB 134–138 antibodies detected exogenous fibrils in the PFF-treated hippocampal (Supplementary Fig. 3a) and cortical (Supplementary Fig. 3b) aSyn KO neurons by ICC. By WB, bands specific to exogenously added fibrils were revealed between 10–35 kDa by these antibodies (Supplementary Fig. 5a, green arrows) except for the 6B2-D12 (126–132) antibody, which failed to detect any bands in the PFF-added neuronal fractions. With the aSyn PTM antibodies, no positivity was detected in the PFF-treated hippocampal or cortical aSyn KO neurons using the aSyn pY39, pS129, pY133, or pY136 antibodies by ICC (Supplementary Fig. 4a, b) or by WB (Supplementary Fig. 5b). Similarly, no positivity was revealed with the LASH-EGT nY39 antibody in the hippocampal or cortical aSyn KO neurons by ICC (Supplementary Fig. 4a, b) or WB (Supplementary Fig. 5b). On the contrary, the monoclonal nY39 antibodies 5E1-G8 and 5E1-C10 were positive both in the PFF-treated and control (PBS-treated) aSyn KO neurons by ICC, signals which we presumed to be non-specific (Supplementary Fig. 4a, b, red arrows). Intriguingly, 6A3-E9 antibody against aSyn-120 showed no background in PBS-treated control neurons but was positive in PFF-treated hippocampal and cortical aSyn KO neurons by ICC (Supplementary Fig. 4a, b, green arrows). Therefore, we cannot rule out the possibility that this antibody may be reactive to aSyn full-length mouse fibrils. By WB, on the other hand, no bands were revealed in the PFF-treated aSyn KO hippocampal and cortical soluble and insoluble fractions (Supplementary Fig. 5b). Collectively, these results confirm the specificity of our antibodies, and suggest that the internalized PFFs do not undergo any type of modifications except for N- and C-terminal cleavages, in the absence of seeding.

**Table 4:** A summary of the mouse aSyn-reactive antibody detection patterns of exogenous PFFs in aSyn KO neurons, newly formed aggregates in PFF-seeded WT neurons and mouse brain tissues.

Antibody	Epitope	aSyn KO neurons		PFF seeding model: WT primary neurons		PFF seeding model: WT mouse brain tissue	
		PFF detection - ICC	PFF detection - WB	Positivity	aSyn pS129* overlap	Positivity	aSyn pS129* overlap
LASH-EGT403	1–5	+	+	+	–	–	–
5B10-A12	1–10	+	+	+	+	+	+
LASH-EGTNter	1–20	+	+	+	+	+	+
LASH-BL 34–45	34–45	+	+	+	+	+	–
LASH-BL 80–96	80–96	+	+	+	–	+	–
BD SYN-1	91–99	+	+	+	–	+	–
2F10-E12	110–115	+	+	+	–	+	+
6B2-D12	126–132	+	–	+	–	+	–
AB 134–138	134–138	+	+	+	+	+	+
LASH-EGT pY39	pY39	–	–	+	–	+	–
AB EP1536Y	pS129	–	–	+	+	+	+
LASH-EGT pS129	pS129	–	–	+	+	+	+
AB pY133	pY133	–	–	+	–	–	–
AB pY136	pY136	–	–	+	–	–	–
LASH-EGT nY39	nY39	–	–	–	–	–	–
5E1-G8	nY39	+ (non-specific)	–	+ (non-specific)	–	–	–
5E1-C10	nY39	+ (non-specific)	–	+ (non-specific)	–	–	–

*aSyn* alpha-synuclein, *ICC* immunocytochemistry, *KO* knockout, *PFF* pre-formed fibril, *WB* Western blot, WT wild-type.

*aSyn pS129 revealed by BL 81A mouse monoclonal and AB MJF-R13 rabbit monoclonal antibodies.

**Fig. 6:**
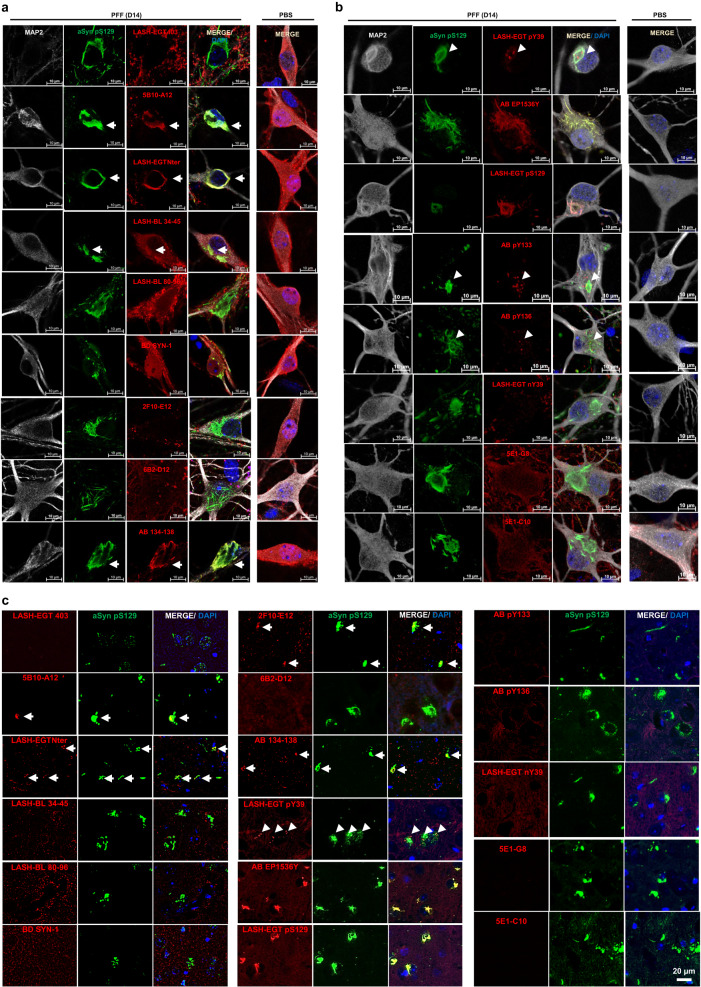
Application of the aSyn antibodies to the cellular and animal seeding models to profile the newly formed aSyn aggregates. WT hippocampal neurons were seeded with PFFs for 14 days, and the newly formed aggregates monitored by ICC using the mouse-reactive **a** non-modified aSyn and **b** aSyn PTM antibodies in parallel to aSyn pS129 antibodies BL 81A or AB MJF-R13. **c** The same type of screening was run in PFF-injected mouse amygdala tissues by IHC. The non-modified aSyn antibody signals overlapping with the aSyn pS129-positive aggregates are marked with an arrow. The punctate positivity shown by aSyn pY39, pY133, and pY136 antibodies in close proximity to aSyn pS129-positive aggregates are shown by arrowheads. Note the non-specific diffuse positivity revealed by the two monoclonal nY39 antibodies 5E1-G8 and 5E1-C10 in the WT hippocampal neurons are also revealed in the aSyn KO neurons using these two antibodies (Supplementary Fig. 4A, B). aSyn alpha-synuclein, DAPI 4’,6-diamidino-2-phenylindole, ICC immunocytochemistry, IHC immunohistochemistry, KO knockout, MAP2 microtubule-associated protein 2, PBS phosphate-buffered saline, PFF pre-formed fibril, PTM post-translational modification, WT wild-type.

Finally, the mouse aSyn-reactive antibodies (Tables 1 and 4) were used to characterize the PTM profile of newly formed aSyn aggregates in the neuronal seeding model^20,23,117^, and in the PFF-injected in vivo model^18,111,118^ of aSyn. Whilst all the non-modified aSyn antibodies detected the endogenous aSyn in PFF-treated or control (PBS-treated) WT hippocampal neurons by ICC, the N-terminal 5B10-A12 (1–10), LASH-EGTNter (1–20), LASH-BL 34–45 and the C-terminal AB 134–138 antibodies showed almost complete overlap with the aSyn pS129-positive inclusions in the PFF-treated neurons (Table 4 and Fig. 6a, arrows), suggesting that these could be useful and alternative tools to aSyn pS129 for monitoring the aSyn aggregation in cell culture, especially if cross-reactivity of the pS129 antibodies is a confounding factor. A similar pattern of overlap with the aSyn-positive inclusions, specifically for the N-terminal 5B10-A12 (1–10), LASH-EGTNter (1–20), the C-terminal 2F10-E12 (110–115) and AB 134–138 antibodies (Table 4 and Fig. 6c, arrows), was seen in the amygdala of WT mice that had been injected with PFFs in the striatum. The amygdala is particularly prone to develop early and substantial aSyn pathology in this model^111^. We speculate that this staining pattern may be due to the preferential exposure of epitopes in the extreme N- and C-terminal aSyn, whereas the hydrophobic NAC region is less accessible and buried in the core of the newly formed aggregates.

With the aSyn nY39 antibodies, we did not see any positivity in the neurons or in mouse tissue (Table 4 and Fig. 6b, c), except for diffuse cytoplasmic staining with 5E1-G8 and 5E1-C10, which we also observed in aSyn KO neurons and therefore deemed non-specific (Supplementary Fig. 4a, b). With the N-terminal tyrosine phosphorylation at Y39, on the other hand, both in the PFF-treated neurons and in the PFF-injected mouse amygdala, we detected punctate structures in close proximity to, and partially overlapping with, the aSyn pS129-positive inclusions (Table 4 and Fig. 6b, c, arrowheads). We noted similar punctate positivity also with aSyn pY133 and pY136 antibodies in the neuronal seeding model (Table 4 and Fig. 6b, arrowheads), which again partially overlapped with the aSyn pS129-positive accumulations. As anticipated, there was a substantial overlap between the signals of the aSyn pS129 antibody BL 81A, which was used to detect the inclusions in the neuronal and mouse models, and those of EP1536Y and LASH-EGT pS129 (Fig. 6b, c). Altogether, these data suggest that aSyn may become hyperphosphorylated during the aggregation and inclusion maturation processes. Further research is needed to decipher the mechanism of formation and fate of these punctate structures. In this study, we have demonstrated the effectiveness of the antibodies we introduced in examining how PTMs dominate aSyn seeding and inclusion formation. In addition, our antibodies enable us to capture the diverse structural and chemical characteristics of aSyn aggregates in cellular and in vivo models of aSyn seeding.

## Discussion

Previous studies have used multiple antibodies to understand the diverse manifestations of aSyn in LBDs and MSA^41–43,45,46,48,49,79,96,119–122^. In this study, we developed, characterized, and validated 12 novel aSyn antibodies targeting the entire sequence and several disease-associated PTMs of aSyn. These antibodies were combined with existing ones to ensure comprehensive coverage of the diverse biochemical and structural aspects of aSyn. A total of 31 antibodies were rigorously assessed using a stringent validation process (Fig. 1d). Among them, we selected 14 antibodies to examine aSyn pathology in the same set of LBD cases (Fig. 5a and Table 3). While all antibodies targeting non-modified aSyn sequences effectively detected LBs, considerable differences were observed, particularly in the identification of astroglial aSyn accumulations, punctate neuronal cytoplasmic inclusions, a subset of LNs and neuropil dots in the substantia nigra and cingulate cortex of LBDs. These findings indicate that individual antibodies have varying capacities to reveal specific aspects of aSyn pathology, which has important implications for disease staging and characterization of different LBD subtypes.

LBs are mature inclusions consisting of a diverse range of aSyn species, including full-length, N- and C-terminally truncated, and other modified forms with different conformations^90,91,95^. Consequently, aSyn antibodies targeting different epitopes throughout its sequence are more likely to detect LBs. However, specific forms or conformations of aSyn in other types of accumulations may be challenging to detect using single antibodies. These include less mature and poorly understood manifestations of aSyn pathology, such as punctate neuronal inclusions, astroglial aSyn accumulations, and a subset of LNs, potentially composed of uniform aSyn proteoforms. By employing an expanded toolbox, we can now systematically analyze aSyn species at the single-cell level using ICC/IHC. Through this approach, we have identified antibodies capable of detecting specific aSyn species and inclusions in different cell types. This advancement allows for future investigations into the factors influencing aSyn pathology formation and the exploration of cellular and molecular processes associated with neurodegeneration.

Our study focused on investigating the most prominent disease-associated PTMs of aSyn, including phosphorylation, nitration, and truncation, in a consistent manner across a set of LB cases. We confirmed that aSyn pS129, a phosphorylation modification, is highly abundant in the substantia nigra, which aligns with previous mass spectrometry studies^29,50^. Importantly, we discovered that LB diseases also exhibit high levels of N-terminal nitration at Y39, a finding not categorically explored due to the absence of specific antibodies^99^. In addition, we observed the widespread presence of N-terminal and C-terminal tyrosine phosphorylations, which have been studied to a limited extent in previous research^68,69,71^. These modifications were found in both neurons and glia across LBDs. However, it is worth noting that our study did not investigate other modifications such as ubiquitination, acetylation, or N- and C-terminal truncations, except for aSyn-120, due to the lack of appropriate antibodies. Future research efforts aimed at developing tools to fill these gaps will provide a more complete picture of aSyn heterogeneity. While our study primarily focused on sporadic and familial LBDs, ongoing studies are underway to explore aSyn pathology in MSA using the same tools.

The neuronal seeding model has provided valuable insights into aSyn aggregation and LB-like inclusion formation, with a focus on aSyn phosphorylation at Serine 129^20,21,23,117^. However, the occurrence of other disease-associated PTMs in these models has not been systematically assessed, except for certain C-terminally cleaved aSyn species^18–21,117,123^. As a result, our understanding of how PTMs influence aSyn seeding and the inclusion formation in these models remains incomplete. In this study, we conducted the first comprehensive profiling of post-translationally modified aSyn species in this model. We found hyperphosphorylation of neuronal aSyn accumulations and identified the presence of punctate intracellular structures with tyrosine phosphorylations (pY39, pY133, pY136) that are not detected by aSyn pS129 antibodies. These Y39-phosphorylated aSyn structures are particularly reminiscent of the pY39-positive neuronal punctae revealed in postmortem LBD tissues (Table 3). Whether these structures represent a subset of vesicular aSyn species with a specific PTM profile and conformation remains unclear but is worthy of further investigation. Therefore, exploring the cellular mechanisms underlying these modifications and their impact on aSyn aggregation, aggregate maturation and aSyn-mediated toxicity holds great translational value.

We offer a carefully curated toolset of antibodies that expands our ability to assess the diversity of aSyn pathology. Although several studies have reported on the development of conformation-specific aSyn antibodies^41,47,48,124^, a comparative analysis of 17 conformational antibodies showed that none could distinguish aSyn oligomers from fibrils^110^. In line with these findings, most of the antibodies reported here recognize monomeric, oligomeric, and fibrillar aSyn forms. This challenge, combined with the need to build antibodies that can specifically target aSyn PTMs, motivated our study to focus on sequential instead of conformational antibody development. While not every study may require the complete set, the detailed characterization of each antibody allows for informed selection based on specific study objectives. Moreover, employing a well-chosen subgroup of these antibodies in staging studies of PD and other LBDs has the potential to address inconsistencies in correlating pathology with symptomatology^125–129^.

By capturing a wide range of aSyn species, we have a valuable opportunity to systematically investigate the correlation between aSyn aggregation, neuronal dysfunction and neurodegeneration in PD and other synucleinopathies. For instance, previous studies examining the absence of LBs in PD patients with leucine-rich repeat kinase 2 (*LRRK2)* mutations have often relied on a limited number of antibodies targeting the C-terminus of aSyn, potentially overlooking other proteoforms of aSyn^130–139^. By re-examining aSyn pathology using our antibody toolbox, we can conclusively determine whether LBs or other forms of aSyn pathology are genuinely absent in these cases. In addition, recent findings from our group indicate that certain PTMs, such as O-linked-N-acetylglucosaminylation (O-GlcNAcylation) and nitration, reduce the seeding activity of aSyn fibrils in primary cultures and in vivo models^140,141^. This suggests that the patterns of PTMs, rather than just the presence of aSyn aggregates, may play a crucial role in determining some of the pathogenic properties of aSyn. Consequently, there is a pressing need for validated tools and methods to accurately map and quantitatively assess the distribution of aSyn PTMs in different brain regions at various stages of disease progression.

In conclusion, we propose that future research should focus on three key areas. Firstly, there is a need to re-evaluate the staging of PD and other LBDs in the central nervous system using large cohorts. Secondly, investigating aSyn aggregation in peripheral tissues using expanded toolsets such as the one described here is likely to offer new insights into aSyn pathology spreading. Thirdly, further characterizing the nature and modifications of aSyn inclusions formed in different cellular and in vivo models of PD using these tools would help us recognize how closely we can replicate human pathology in these model systems. These endeavors would allow us to better understand the key determinants of aSyn pathology formation, disease-spreading pathways and neurodegeneration.

## Methods

### Generation of aSyn monoclonal antibodies

A combination of aSyn human recombinant proteins and peptides against different forms and modifications of aSyn was used for the immunization of BALB/c mice (Supplementary Fig. 10 and Supplementary Table 1). These were solubilized in PBS and, where appropriate, conjugated to the carrier protein keyhole limpet hemocyanin (KLH). Following pre-immune serum collection, BALB/c mice were subcutaneously injected with the immunogen-complete Freund’s adjuvant (CFA) mixture on Day 0, and with immunogen-incomplete Freund’s adjuvant (IFA) on Day 21 and Day 42. Test bleeds were run on Days 35 and 56, and antibody response was evaluated by ELISA, WB and DB. Animals with strong immunoreactivities were euthanized and their splenocytes surgically harvested.

Hybridoma technology was used for the production of monoclonal antibodies, where the antibody-producing lymphocytes were fused with Sp2/0-Ag14 (ATCC #CRL-8287) mouse myeloma cells using polyethylene glycol (PEG) at a 5:1 ratio. The hybridomas were grown in hypoxanthine-aminopterin-thymidine (HAT) selective media to eliminate unfused myeloma cells. Supernatants of 6 to 44 clones per program were tested by ELISA, DB and WB against a selected library of aSyn proteins (Supplementary Table 2) to determine the clones with the strongest and the most specific results. Selected clones were further sub-cultured for several rounds to maintain stability, subjected to serial dilution to ensure monoclonality, and screened by ELISA, DB and WB for the identification of positive and specific clones. Isotyping and in vitro production of antibodies were carried out with the selected subclones, on an Akta 25 FPLC system (RRID:SCR_023461) using a 25 mL protein G sepharose column (Cytiva) according to the instructions of the manufacturer. Briefly, the resin was equilibrated using 10 column volumes (CV) of buffer A (20 mM phosphate buffer pH 7.2) before loading the filtrated sample on the column. Following a wash step of 10 CV with buffer A, antibodies were eluted using an isocratic elution with buffer B (100 mM glycine pH 2.7) and were immediately pH-neutralized with 1 M Tris buffer pH 8.0 upon their harvest in fractionation tubes. Buffer exchange was performed against 10 mM PBS using a 30 kDa dialysis membrane, and the harvested antibodies were stored at −70 °C. Antibody concentrations were determined using absorbance reading at 280 nm, and purity was determined by size exclusion chromatography. An average of 45 mg from each of the 12 antibodies were obtained.

### Expression and purification of recombinant proteins

The expression and purification of human and mouse aSyn were carried out as described^142^. Briefly, pT7-7 plasmids encoding variants of mouse and human aSyn were used to transform BL21(DE3) chemically competent *E. coli* and let to grow on an agar dish with ampicillin. One colony was transferred to 400 mL of Luria broth media with ampicillin at 100 µg/mL and left to grow at 37 °C on a shaker at 180RPM for 16 h. The small culture was then used to inoculate a 6 L culture with ampicillin at 100 µg/mL. At an optic density (OD_600_) of 0.5–0.6, isopropyl β-D-1-thiogalactopyranoside was added at a final concentration of 1 mM to induce aSyn expression. The large culture was left to grow further for 4 h on the shaker, centrifuged at 4000× *g* for 15 min at 4 °C. The pellet was re-suspended on ice in lysis buffer (10 mL p/L of culture) containing 20 mM Tris pH 8.0, 0.3 mM phenylmethylsulfonyl fluoride (PMSF) protease inhibitor and cOmplete, mini, EDTA-free protease inhibitor cocktail tablet (Roche #4693159001; one tablet per 10 mL lysis buffer). Cells were lysed by sonication (59s-pulse and 59s-no pulse over 5 min at 60% amplitude). The lysate was centrifuged at 4 °C for 30 min at 20,000× *g*, the supernatant boiled for 5–15 min at 100 °C, and the centrifugation step was repeated. The supernatant was filtered using a 0.22 µm syringe filter and purified via anion exchange chromatography and reverse-phase high-performance liquid chromatography (HPLC). The quality control of the proteins was run via analysis by liquid chromatography-mass spectrometry (LC-MS), ultra-performance liquid chromatography (UPLC) and SDS-PAGE separation and Coomassie staining. aSyn nY39, pY39, pS87, pY125, and pS129 protein standards were prepared using semi-synthesis as previously described^143^. Recombinant gSyn was purchased from Abcam (#ab48712). Tau 1N4R^144^, a-beta 42^145^, and TDP-43^146^ were expressed and purified as described.

### Generation of aSyn oligomers

Generation of aSyn oligomers was carried out as previously described^110^. Briefly, aSyn human WT monomers were dissolved in PBS for a final concentration of 12 mg/mL and was supplemented with benzonase at 1 µL/mL. The solution was filtered (0.22 µm filter unit; Merck-Millipore #SLGP033RS), incubated in low-protein binding tubes at 37 °C for 5 h at 900 RPM and centrifuged for 10 min at 12,000× *g* at 4 °C to remove any insoluble aSyn aggregates. In total, 5 mL of supernatant was run through a PBS-equilibrated Hiload 26/600 Superdex 200 pg column (Sigma-Aldrich #28-9893-36). The protein elution was monitored by ultraviolet (UV) absorbance at 280 nm. Eluted fractions were screened via SDS-PAGE. Oligomeric fractions were characterized by EM, circular dichroism (CD) before being collected, snap frozen and stored at −20 °C.

### Generation of aSyn pre-formed fibrils

Lyophilized human or mouse aSyn WT monomers were re-suspended in PBS for a final concentration of 2–4 mg/mL, and the pH was adjusted to 7.5. The protein solution was passed through filters with 100 kDa cut-off to remove any spontaneously formed aggregates. Protein concentration was measured via UV absorption at 280 nm and/or by bicinchoninic acid (BCA) assay on a Tecan Infinite M200 microplate reader (RRID:SCR_019033). Monomers in solution were left on an orbital shaker (at 1000 RPM) for 5 days at 37 °C. For application to cellular seeding models, the fibrils were sonicated to achieve a median fibril length of 50–100 nm. The final fibril preparation was characterized for the monomer-to-fibril ratio by sedimentation and filtration assays as described in ref. ^147^, for amyloid formation by Thioflavin T (ThT) assay, and for fibril length quantification by electron microscopy analysis.

### Dot/slot blot and Western blot analyses using aSyn recombinant proteins

All blots were processed in parallel and derived from the same experiments. For the DB analysis, aSyn proteins were diluted in PBS and blotted on a nitrocellulose membrane of 0.22 µm in 100 µL volume corresponding to 200 ng of protein loading (unless indicated otherwise). For the WB analysis, aSyn proteins were diluted in PBS and Laemmli buffer 4× (50% glycerol, 1 M Tris at pH 6.8, 20% β-mercaptoethanol, 10% SDS and 0.1% bromophenol blue), loaded onto a 4–16% Tricine gel in 10 µL volume corresponding to 100 ng of protein loading and transferred onto a nitrocellulose membrane of pore size 0.22 µm using a semi-dry transfer system (BioRad) for 45 min at 0.5 A and 25 V. Where appropriate, Ponceau S staining (2% Ponceau S in 5% acetic acid) was applied as a protein loading control. The membranes were blocked overnight at 4 °C in Odyssey blocking buffer (Li-Cor). They were incubated with primary antibodies diluted in PBS for 2 h at room temperature (RT), washed three times for 10 min in PBS with 0.01% Tween-20 (PBS-T), incubated in dark with secondary antibodies diluted in PBS and washed three times for 10 min in PBS-T. For the primary and secondary antibody details, see Supplementary Table 4. The membranes were imaged at 700 nm and/or 800 nm using the Li-Cor Odyssey CLx imaging system (RRID:SCR_014579), and the images were processed using Image Studio Lite (RRID:SCR_013715).

### Surface plasmon resonance

SPR data were collected on a Biacore 8 K device (GE Healthcare #29215379). Antibody (6A3) was immobilized on a CM5 biosensor chip (GE Healthcare) at 10–20 μg/mL concentration in 10 mM acetate solution (GE Healthcare) at pH 4.5 to reach a final surface ligand density of around 2000–4000 response units (RUs). In short, the whole immobilization procedure using solutions of 1-ethyl-3-(3-dimethyl aminopropyl) carbodiimide (EDC) and N-hydroxy succinimide (NHS) mixture, antibody sample and ethanolamine, was carried out at a flow rate of 10 μL/min into the flow cells of the Biacore chip. The carboxyl groups on the sensorchip surface were activated by injecting 200 μL of 1:1 (v/v) mixture of EDC/NHS (included in the amine coupling kit, Cytiva Life Sciences) into both flow cells 1 and 2 and followed by the injection of antibodies overflow cell 2 for 180 s. The remaining activated groups in both the flow cells were blocked by injecting 129 μL of 1 M ethanolamine-HCl pH 8.5. The sensor chip coated with antibodies were equilibrated with PBS buffer before the initiation of the binding assays. Serial dilutions of analytes such as aSyn monomers (human WT 1–20 or human WT 1–140) at a concentration ranging between 2.5 μM to 0.1 μM in PBS buffer were injected into both flow cells at a flow rate of 30 μL/min at 25 °C. Each sample cycle has the contact time (association phase) of 120 s and is followed by a dissociation time of 600 s. After every injection cycle, surface regeneration of the Biacore chips was performed using 10 mM glycine (pH 3.0).

### Mouse primary neuronal culture and seeding assay

Primary hippocampal and cortical neurons were collected from P0 pups of WT (C57BL/6J-RccHsd, Harlan) or aSyn KO (C57BL/6J-OlaHsd, Harlan) mice, according to the dissection procedure described elsewhere^148^. Following the plating in poly-l-lysine-coated plates (300,000 cells/mL), the neurons were left to mature at 37 °C with 5% CO_2_. WT neurons were treated with 70 nM aSyn mouse WT PFFs on day in vitro (DIV)7 and left to incubate for 14 days; and aSyn KO neurons on DIV20 and left to incubate for 24 h, as described^20,21,23,117^.

### Immunocytochemistry and confocal imaging

Mouse primary neurons were washed twice in PBS, fixed with 4% PFA for 20 min at RT, and stained as described elsewhere^149^. The antibodies used for ICC are detailed in Supplementary Table 4. Imaging was carried out on a confocal laser-scanning microscope (Zeiss LSM 700, RRID:SCR_017377) and image analysis on Zen Digital Imaging software (RRID: SCR_013672).

### Cell lysis, sequential extraction, and Western blotting

Mouse primary neurons were washed in PBS twice on ice and extracted in Tris-buffered saline (TBS; 50 mM Tris and 150 mM NaCl at pH7.5) with 1% Triton X-100 (Tx-100) and with protease inhibitor (PI) cocktail (1:100), 1 mM phenylmethane sulfonyl fluoride (PMSF), phosphatase inhibitor cocktails 2 and 3 (1:100), as described previously^20,21,23,117^. The cells were lysed via sonication (1 s intermittent pulse 10 times at 20% amplitude) using a small probe (Sonic Vibra Cell, Blanc Labo, Switzerland). The lysate was incubated for 30 min on ice and centrifuged at 100,000× *g* for 30 min at 4 °C. The supernatant i.e., the “soluble” fraction, was collected and diluted in 4× Laemmli buffer. As the wash step, the pellet was re-suspended in lysis buffer, sonicated and centrifuged as described above. The supernatant was discarded and the pellet re-suspended in TBS with 2% sodium dodecyl sulfate supplemented with protease and phosphatase inhibitors as described above. The re-suspension, i.e., the “insoluble fraction”, was sonicated (1 s intermittent pulse 15 times at 20% amplitude) and diluted in 4× Laemmli buffer. Protein concentration was determined by BCA assay separately for the soluble and the insoluble fractions.

The soluble and insoluble fractions were separated on a 16% Tricine gel (ThermoFisher), and transferred onto a nitrocellulose membrane of pore size 0.22 µm using a semi-dry transfer system (BioRad) for 45 min at 0.5 A and 25 V. The membranes were blocked overnight at 4 °C in Odyssey blocking buffer (Li-Cor) and washed three times for 10 min in PBS with 0.01% Tween-20 (PBS-T). Membranes were incubated with primary antibodies diluted in PBS for 2 h at RT, washed three times for 10 min in PBS-T, incubated in dark with secondary antibodies diluted in PBS and washed three times for 10 min in PBS-T. For the antibody details, see Supplementary Table 4. The membranes were imaged at 700 nm and/or 800 nm using the Li-Cor Odyssey CLx imaging system (RRID:SCR_014579), and the images were processed using Image Studio Lite (RRID:SCR_013715).

### Animals and intra-striatal stereotaxic injection procedure

All animal experimentation was performed in compliance with the European Communities Council Directive of 24 November 1986 (86/609EEC) and with approval of the Cantonal Veterinary Authorities (Vaud, Switzerland) and the Swiss Federal Veterinary Office (authorization number VD2067.2). C57BL/6JRj male mice (Janvier Labs) at 3 months of age were stereotaxically injected with aSyn mouse WT PFFs (5 mg in 2 mL PBS) in the right dorsal striatum. Six months post-injection, the animals were sacrificed by intracardiac perfusion with heparinized sodium chloride (NaCl; 0.9%) and fixed with 4% PFA in PBS overnight, and paraffin-embedded for immunohistochemical studies. For the antibody validation studies, naïve adult aSyn KO male mice (C57BL/6J-OlaHsd, Harlan) were sacrificed, and brain sections were prepared in the same way as for PFF-injected WT animals.

### Immunofluorescent labeling and imaging of mouse brain tissue

WT and aSyn KO mouse paraffin-embedded brain sections were cut coronally to 4 µm and dewaxed. Epitope retrieval was carried out in 10 mM trisodium citrate buffer at pH 6.0 for 20 min at 95 °C. Tissue blocking was run by incubation in 3% bovine serum albumin (BSA) and 0.1% Tx-100 in PBS for 1 h at RT. Sections were then incubated with the primary antibody solution overnight at 4 °C, and with the secondary antibody solution for 60 min at RT (Supplementary Table 4). Slides were mounted using an aqueous mounting medium, and tiled imaging was carried out on the Olympus VS120 microscope (RRID:SCR_018411).

### Human brain tissue samples

The cases selected from the Queen Square Brain Bank (QSBB), University College London Institute of Neurology in London, and Oxford Brain Bank (OBB), Nuffield Department of Clinical Neurosciences in the University of Oxford, were collected in accordance with approved protocols by the London Multicentre Research Ethics Committee and the Ethics Committee of the University of Oxford (ref 15/SC/0639). All donors had given written informed consent prior to the brain collection. The samples were stored under the license approved by the Human Tissue Authority (HTA; license numbers 12198 for QSBB and 12217 for OBB). The ethical approval for the research was given by the National Research Ethics Service (NRES) Committee London Central. Case demographics are detailed in Table 5.

**Table 5:** Demographics of the human postmortem cases included in this study.

Case ID	Sex (m/f)	Age at diagnosis (y)	Age at death (y)	Disease duration (y)	Postmortem delay (h)	Braak stage (LB-type pathology)	Braak stage (AD-type pathology)
PD1	f	65	86	21.0	78.4	6	1
PD2	m	55	75	20.0	88.5	6	1
PD3	m	60	84	24.0	71.0	6	2
PD4	m	62	80	18.5	67.0	6	3
PD5	m	68	80	11.9	100.0	6	2
PD6	f	61	81	20.0	135.0	6	2
PD7	m	80	89	8.5	26.0	6	4
PDD1	m	77	92	14.0	41.0	6	3
PDD2	m	61	80	19.0	66.0	6	2
DLB1	f	78	89	11.4	63.0	6	3
*SNCA* G51D1	m	19	49	30.0	43.0	na	na
*SNCA* G51D2	f	69	75	6.5	62.0	na	1
*SNCA* G51D3	m	45	52	6.5	85.3	na	1
*SNCA* H50Q1	f	70	83	12.5	na	na	na
*SNCA* duplication1	m	55	62	6.9	143.0	6	2
AD1	m	54	64	9.8	95.5	0	6
AD2	f	48	64	16.6	70.0	na	6
PiD1	m	55	71	16.4	51.0	0	0
PiD2	m	52	65	13.5	48.0	0	0
FTLD-TDP/C1	f	51	65	14.0	22.0	0	0
FTLD-TDP/C2	m	53	71	17.3	54.0	0	0
PSP1	f	74	79	6.3	105.0	0	2
PSP2	m	68	75	7.0	57.0	0	2
CBD1	m	51	60	9.0	40.0	na	0
CBD2	m	60	65	5.0	50.0	0	2
CTR1	f	67	84	16.0	40.0	0	2
CTR2	m	63	80	16.6	11.0	0	2
CTR3	f	na	89	na	24	0	2
CTR4	m	na	80	na	48	0	2

*AD* Alzheimer’s disease, *CBD* corticobasal degeneration, *CTR* control, *DLB* dementia with Lewy bodies, *f* female, *FTLD-TDP/C* frontotemporal lobar degeneration of transactive response DNA-binding protein 43 type C, *h* hours, *ID* identity, *LB* Lewy body, *m* male, *na* not available, *PD* Parkinson’s disease, *PDD* Parkinson’s disease with dementia, *PiD* Pick’s disease, *PSP* progressive supranuclear palsy, *y* years.

### Immunohistochemistry of human brain samples with 3,3′-diaminobenzidine (DAB) revelation and imaging

FFPE sections were cut sequentially to 8 µm of thickness and de-paraffinized. The rationale for the selection of the final epitope retrieval approach and dilution for each antibody was to enable the antibodies to reveal the optimal number of pathological inclusions with minimal non-specific background. For epitope retrieval, the sections were treated with 80–100% formic acid for 10 min at RT and/or with citrate buffer (pH 6.0) for 10 min at 121 °C under pressure. Sections were treated with 3% hydrogen peroxide in PBS for 30 min to quench the endogenous peroxidase. After the blocking in 10% fetal bovine serum (FBS) for 30 min, sections were incubated in primary antibody solution overnight at 4 °C. For the primary antibody details and their optimized IHC settings, see Supplementary Tables 4 and 5. After being rinsed in PBS-Tween 0.1% (PBS-T), sections were incubated in the secondary antibody-horseradish peroxidase (HRP) complex from the REAL EnVision detection kit (Dako #K5007) for 1 h at RT. Sections were rinsed in PBS-T before visualization with 3,3’-diaminobenzidine (DAB). They were counterstained with hematoxylin, cleared in xylene, and mounted using distyrene plasticizer xylene (DPX). Imaging was carried out using an Olympus VS120 microscope (RRID:SCR_018411) and image analysis using QuPath (RRID:SCR_018257).

### Immunofluorescent labeling and imaging of human brain tissue

Following the blocking in 3% BSA and 0.3% Tx-100 in PBS for 60 min at RT, sections were washed in PBS for 5 min and incubated for 1 min in TrueBlack lipofuscin autofluorescence quencher (Biotium #23,007) in 70% ethanol. The sections were washed in PBS (3 × 5 min) and incubated in primary antibodies overnight at 4 °C. For the primary antibody details and their optimized IF settings, see Supplementary Tables 4 and 5. After rinsing in PBS, the sections were incubated in secondary antibodies for 1 h at RT in the dark and washed in PBS. Where applicable, the sections were then incubated in biotinylated primary antibody solution at 4 °C overnight, rinsed in PBS, incubated in streptavidin secondary antibody solution for 1 h at RT in the dark, and rinsed in PBS. The slides were mounted using an aqueous mounting medium with DAPI (Vector Laboratories #H-1500-10). Imaging was carried out on a Leica DM5500 B upright microscope (RRID:SCR_020219), and image analysis on Leica Application Suite X (RRID:SCR_013673).

### Reporting summary

Further information on research design is available in the Nature Research Reporting Summary linked to this article.

### Supplementary information

Supporting information

Reporting Summary

## Data Availability

The datasets used and/or analyzed during the current study are available from the corresponding author on request.
