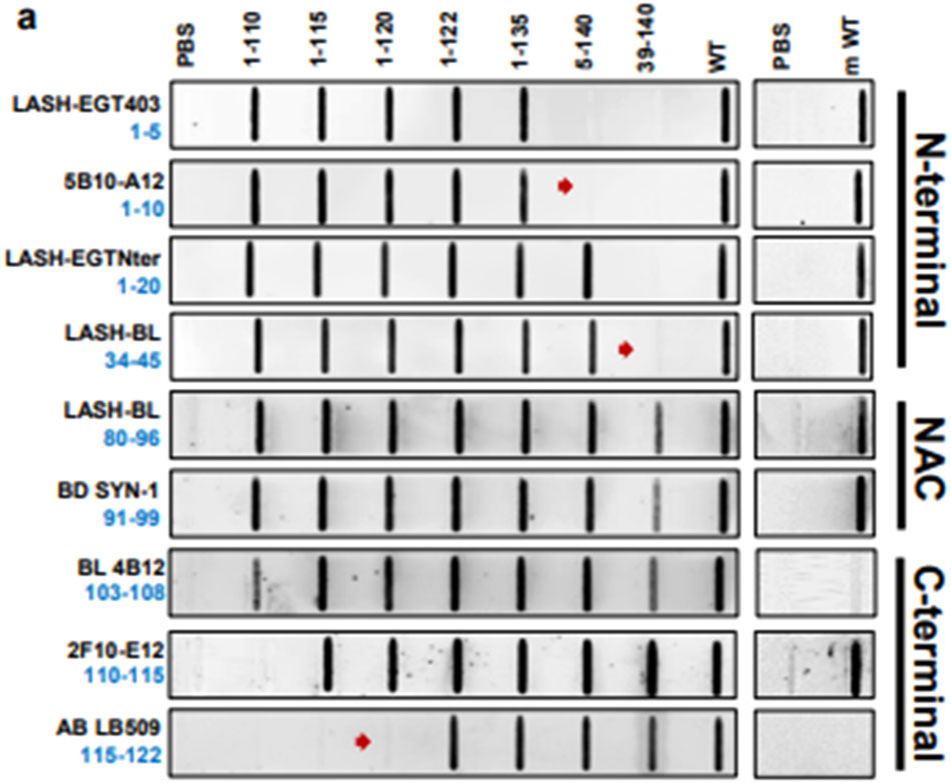# Author Correction: Development and validation of an expanded antibody toolset that captures alpha-synuclein pathological diversity in Lewy body diseases

**DOI:** 10.1038/s41531-024-00634-0

**Published:** 2024-01-11

**Authors:** Melek Firat Altay, Senthil T. Kumar, Johannes Burtscher, Somanath Jagannath, Catherine Strand, Yasuo Miki, Laura Parkkinen, Janice L. Holton, Hilal A. Lashuel

**Affiliations:** 1 Laboratory of Molecular and Chemical Biology of Neurodegeneration, Brain Mind Institute, EPFL, Lausanne, Switzerland; 2 Department of Human Genetics, Inselspital, Bern University Hospital, University of Bern, Bern, Switzerland; 3 Queen Square Brain Bank for Neurological Disorders, University College London Queen Square Institute of Neurology, London, England; 4 Department of Neuropathology, Institute of Brain Science, Hirosaki University Graduate School of Medicine, Hirosaki, 036-8562 Japan; 5 Oxford Parkinson’s Disease Centre, University of Oxford, Oxford, UK; 6 Nuffield Department of Clinical Neurosciences, University of Oxford, Oxford, UK

**Keywords:** Neuroscience, Diseases of the nervous system

Correction to: *npj Parkinson’s Disease* 10.1038/s41531-023-00604-y, published online 07 December 2023

In this article, the wrong slot blots of LASH-BL34-45 and BL 4B12 were presented in Figure 2a; the figure should have appeared as shown below. The original article has been corrected.