# Supplementary material for: Development and validation of an expanded antibody toolset that captures alpha-synuclein pathological diversity in Lewy body diseases
Source: NPJ Parkinsons Dis. 2023 Dec 7;9:161. doi: 10.1038/s41531-023-00604-y (PMC10703845; doi:10.1038/s41531-023-00604-y)
Supplement: Supplementary file 1 — Supporting information [file 41531_2023_604_MOESM1_ESM.pdf]

SUPPLEMENTARY INFORMATION

Development and validation of an expanded antibody toolset that captures alpha-synuclein pathological diversity in Lewy body diseases

Melek Firat Altay[1,2], Senthil T. Kumar[1], Johannes Burtscher[1], Somanath Jagannath[1], Catherine Strand[3], Yasuo Miki[3,6], Laura Parkkinen[4,5], Janice L. Holton[3], Hilal A. Lashuel*[1]

[1] Laboratory of Molecular and Chemical Biology of Neurodegeneration, Brain Mind Institute, EPFL, Lausanne, Switzerland

[2] Department of Human Genetics, Inselspital, Bern University Hospital, University of Bern, Bern, Switzerland

[3] Queen Square Brain Bank for Neurological Disorders, University College London Queen Square Institute of Neurology, London, England

[4] Oxford Parkinson's Disease Centre, University of Oxford, Oxford, UK

[5] Nuffield Department of Clinical Neurosciences, University of Oxford, Oxford, UK

[6] Department of Neuropathology, Institute of Brain Science, Hirosaki University Graduate School of Medicine, Hirosaki 036-8562, Japan

Running title: unmasking alpha-synuclein pathologies in Lewy body diseases

*To whom correspondence should be addressed: Laboratory of Molecular and Chemical Biology of Neurodegeneration, Brain Mind Institute, Ecole Polytechnique Fédérale de Lausanne, 1015 Lausanne. Tel: +41216939691, Fax: +41216939665.

e-mail: hilal.lashuel@epfl.ch

Keywords: alpha-synuclein, Parkinson's disease, Lewy body diseases, post-translational modification, neuropathology, aggregation

SUPPLEMENTARY FIGURES

SUPPLEMENTARY FIGURE 1

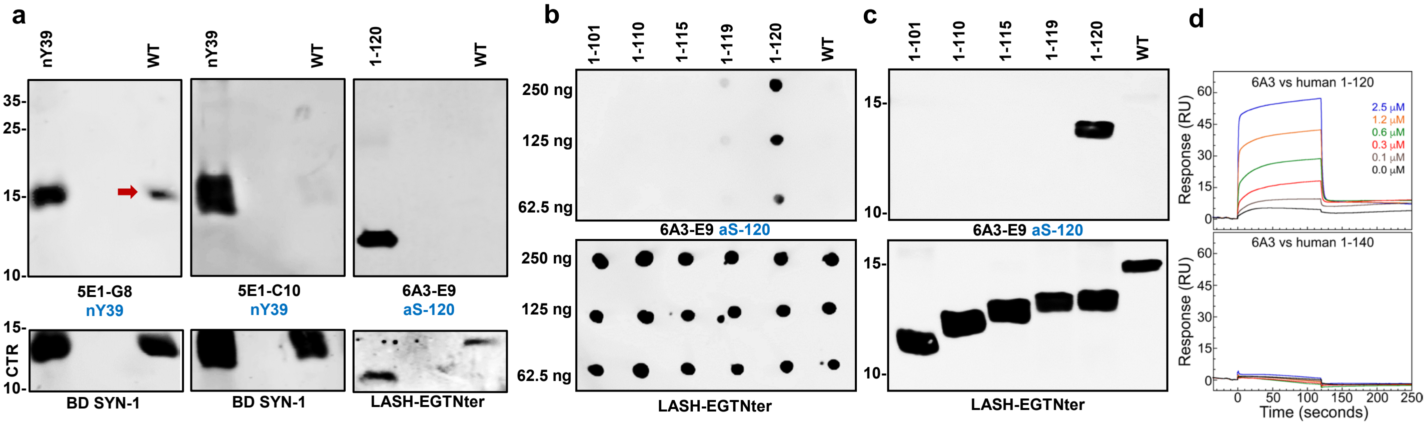

Supplementary Figure 1: Specificity validation of the novel aSyn PTM antibodies using human recombinant aSyn standards. Novel monoclonal aSyn PTM antibodies were validated by **(a)** WB screening. The 5E1-G8 antibody showed non-specific positivity to aSyn WT by WB (red arrow). Further **(b)** DB and **(c)** WB analyses on 6A3-E9 showed that this antibody is specific to human aSyn truncated at residue 120. **(d)** SPR sensograms showed the binding responses of immobilized antibody 6A3-E9 against varying concentrations of aSyn human 1-120 (top) or aSyn human WT (bottom). aSyn = alpha-synuclein; CTR = control; DB = dot/slot blot; PTM = post-translational modification; SPR = surface plasmon resonance; WB = Western blot; WT = wild-type

SUPPLEMENTARY FIGURE 2

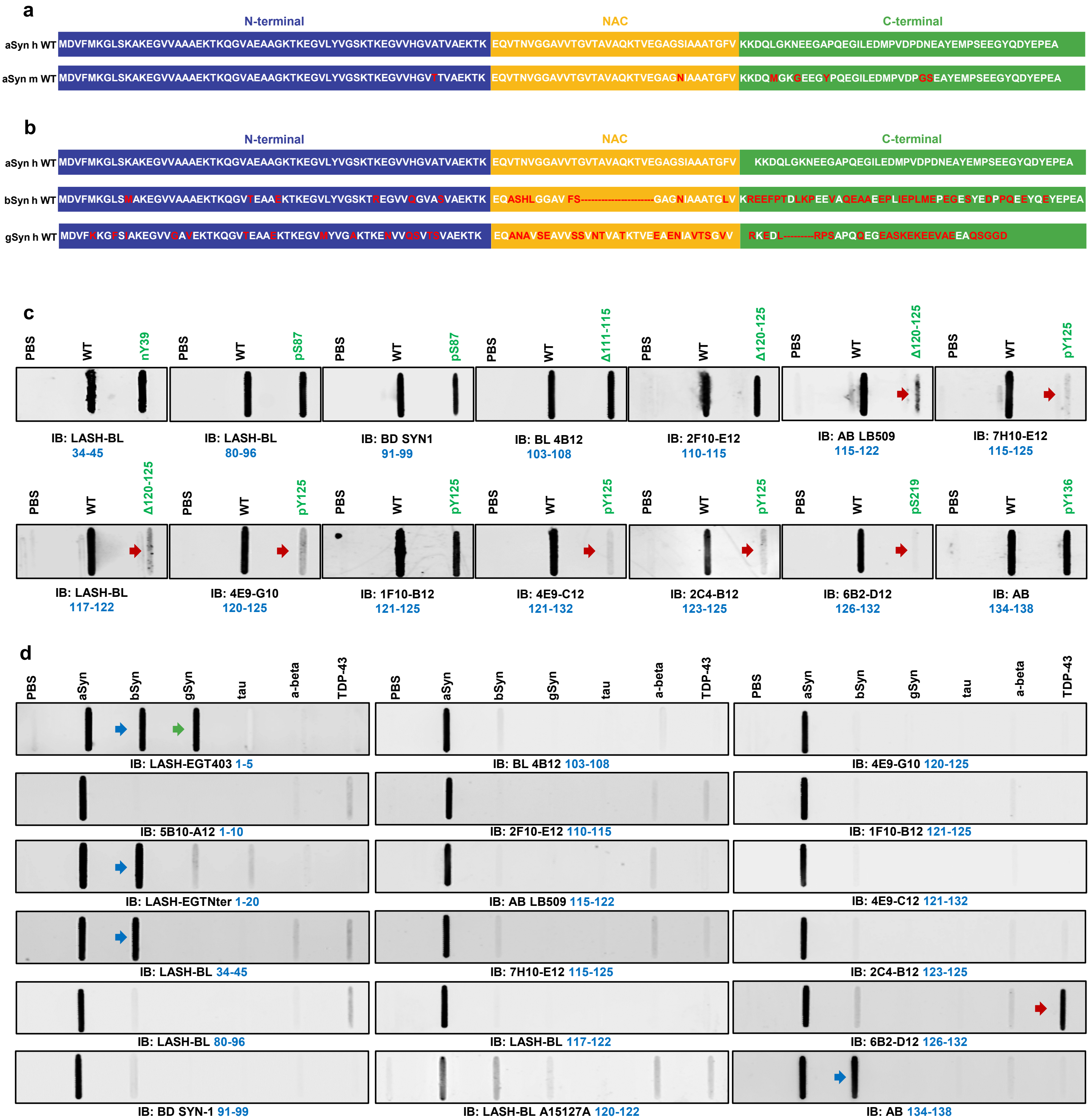

Supplementary Figure 2: aSyn antibody sensitivity to neighboring aSyn PTMs, reactivity to other synuclein family proteins and amyloidogenic proteins. (a) The amino acid sequence of aSyn human and its mouse orthologue. **(b)** The synuclein family comprises three homologous proteins – aSyn, bSyn and gSyn. The residue differences are highlighted in red. **(c)** The sensitivities of non-modified aSyn antibodies to PTMs neighboring or overlapping their epitopes were explored by DB (red arrows). Protein loading control was run via Ponceau S staining. **(d)** Reactivity of the non-modified aSyn antibodies to other members of the synuclein family and to other amyloidogenic proteins tau (1N4R), a-beta 42 and TDP-43 were studied by DB. Protein loading control was run via Ponceau S staining. Blue arrows indicate reactivity to bSyn, green arrows to gSyn and red arrows cross-reactivity to other amyloidogenic proteins. a-beta = amyloid-beta; aSyn = alpha-synuclein; bSyn = beta-synuclein; DB = dot/slot blot; gSyn = gamma-synuclein; h = human; IB = immunoblot; m = mouse; NAC = non-amyloid component; PBS = phosphate buffered saline; PTM = post-translational modification; tau = tubulin-associated unit; TDP-43 = transactive response DNA-binding protein 43kDa; WT = wild-type

SUPPLEMENTARY FIGURE 3

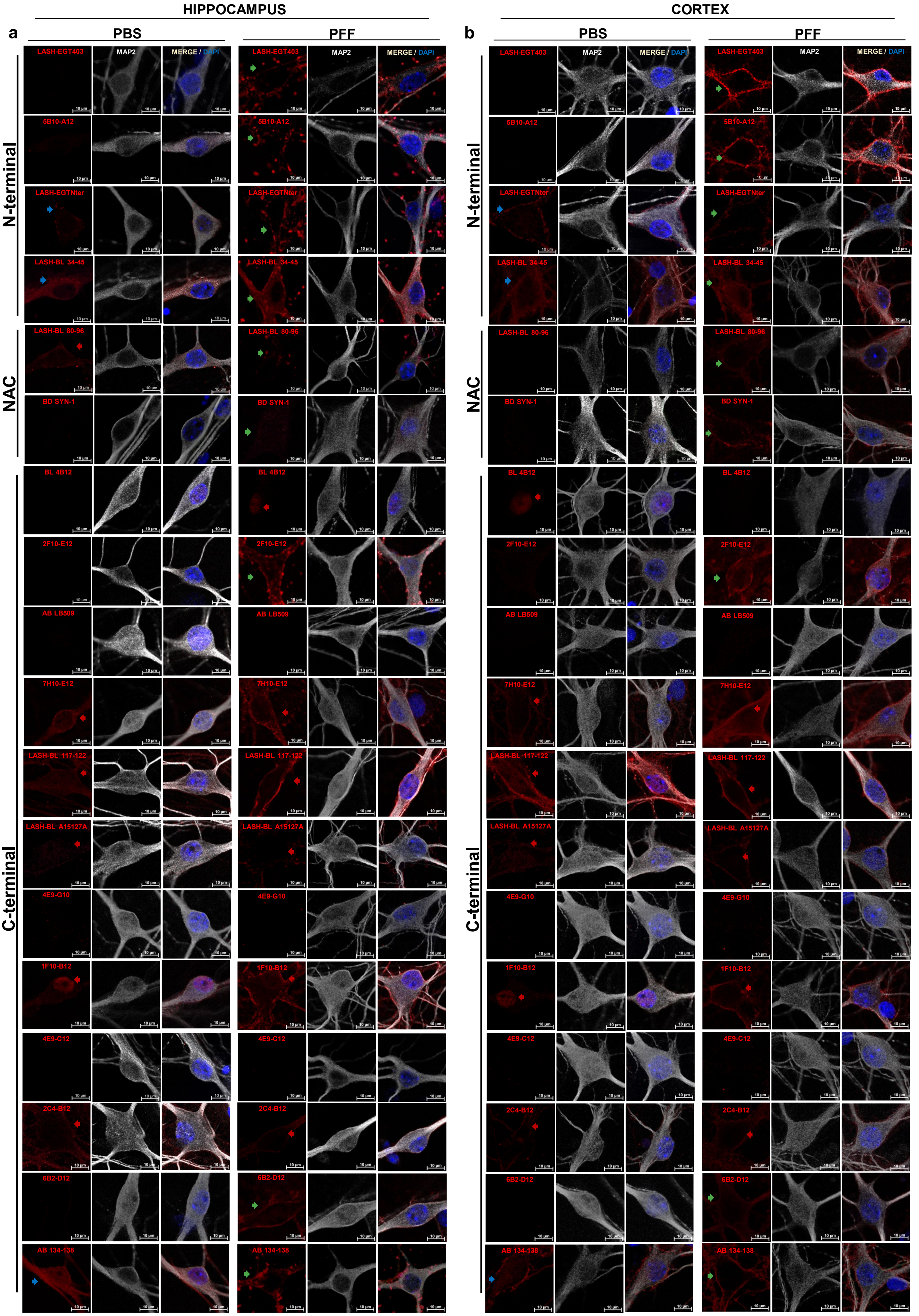

Supplementary Figure 3: Specificity validation of non-modified aSyn antibodies on aSyn KO hippocampal and cortical neurons by ICC. PBS- and PFF-treated aSyn KO **(a)** hippocampal and **(b)** cortical neurons were immunostained to validate the specificity of the antibodies with epitopes against the N-terminus, NAC region and C-terminus of aSyn. Blue arrows indicate bSyn positivity in PBS-treated neurons, green arrows indicate positivity to aSyn mouse WT fibrils in PFF-treated neurons, and red arrows indicate non-specific background both in PBS- and PFF-treated neurons. aSyn = alpha-synuclein; bSyn = beta-synuclein; DAPI = 4',6-diamidino-2-phenylindole; ICC= immunocytochemistry; KO = knockout; NAC = non-amyloid component; MAP2 = microtubule-associated protein 2; PBS = phosphate buffered saline; PFF = pre-formed fibril; WT = wild-type

SUPPLEMENTARY FIGURE 4

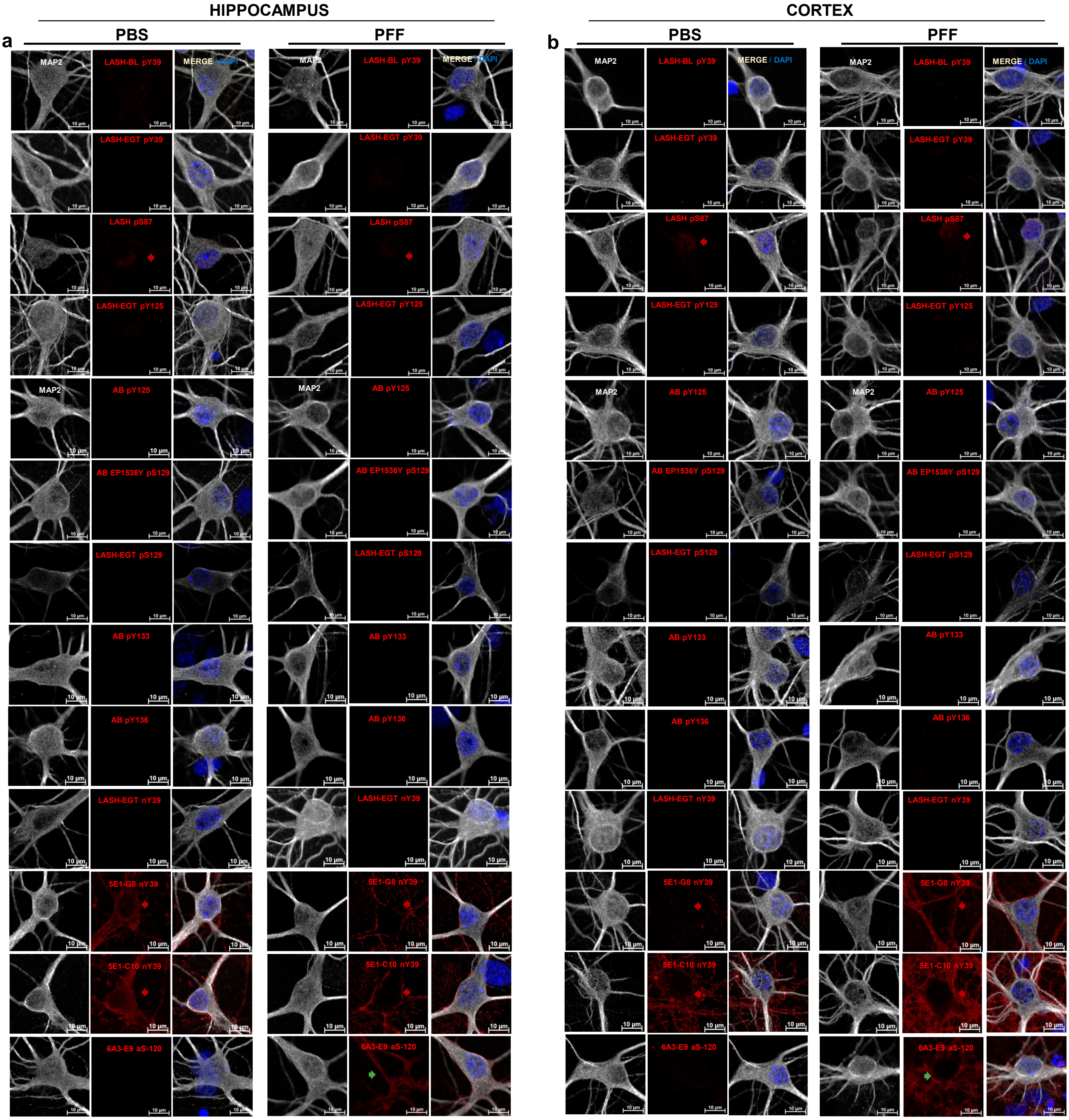

Supplementary Figure 4: Specificity validation of aSyn PTM antibodies on aSyn KO hippocampal and cortical neurons by ICC. PBS- and PFF-treated aSyn KO **(a)** hippocampal and **(b)** cortical neurons were immunostained to validate the specificity of the antibodies with epitopes against the PTMs of aSyn. Green arrows indicate positivity to aSyn mouse WT fibrils in PFF-treated neurons, and red arrows indicate non-specific background both in PBS- and PFF-treated neurons. aSyn = alpha-synuclein; bSyn = beta-synuclein; DAPI = 4',6-diamidino-2-phenylindole; ICC = immunocytochemistry; KO = knockout; MAP2 = microtubule-associated protein 2; PBS = phosphate buffered saline; PFF = pre-formed fibril; PTM = post-translational modification; WT = wild-type

SUPPLEMENTARY FIGURE 5

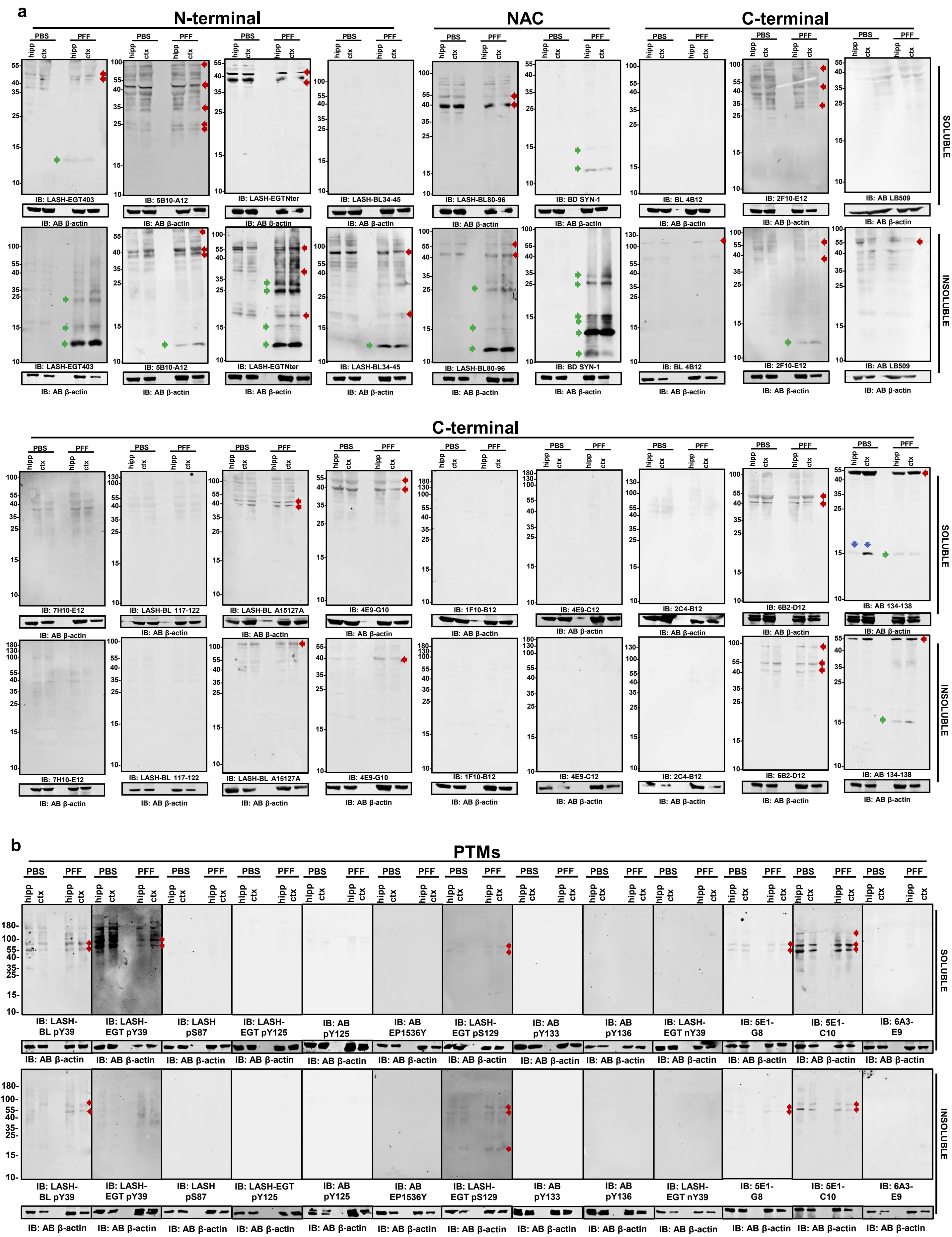

Supplementary Figure 5: Specificity validation of aSyn antibodies on aSyn KO hippocampal and cortical neurons by WB. PBS- and PFF-treated aSyn KO hippocampal and cortical neurons were separated to soluble and insoluble fractions by sequential extraction and stained using **(a)** non-modified and **(b)** aSyn PTM antibodies for specificity validation. Green arrows indicate bands specific to aSyn mouse WT fibrils, blue arrows indicate bSyn-specific bands, and red arrows indicate non-specific background. aSyn = alpha-synuclein; bSyn = beta-synuclein; ctx = cortex; hipp = hippocampus; IB = immunoblot; NAC = non-amyloid component; KO = knockout; PBS = phosphate buffered saline; PFF = pre-formed fibril; PTM = post-translational modification; WB = Western blot; WT = wild-type

SUPPLEMENTARY FIGURE 6

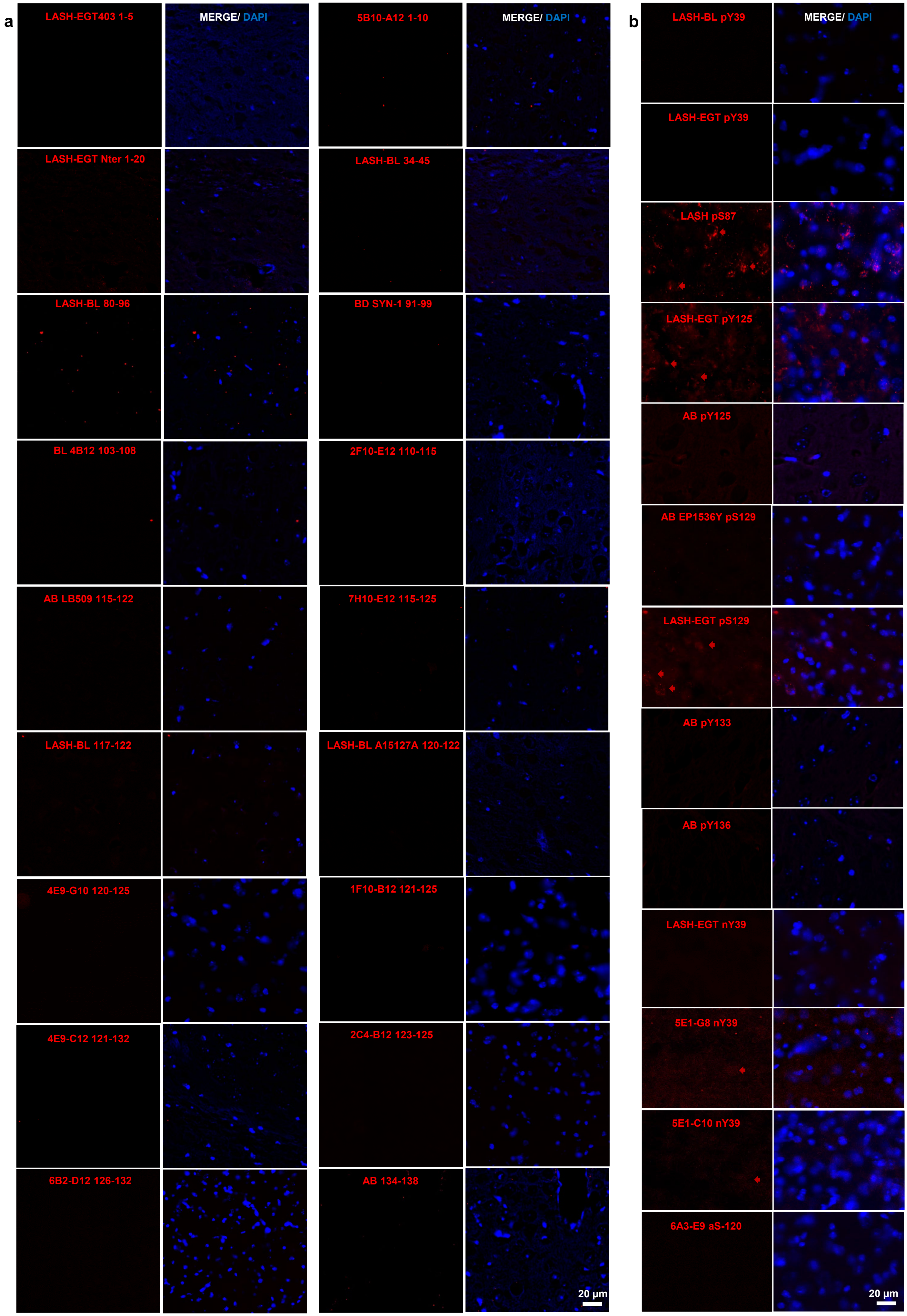

Supplementary Figure 6: Specificity validation of aSyn antibodies on aSyn KO mouse brain tissue by IF. The in-house and commercial antibodies against **(a)** non-modified aSyn and **(b)** aSyn PTMs were screened for their specificity using aSyn KO mouse amygdala sections. Red arrows indicate non-specific background. aSyn = alpha-synuclein; DAPI = 4',6-diamidino-2-phenylindole; IF = immunofluorescence; KO = knockout; PTM = post-translational modification

SUPPLEMENTARY FIGURE 7

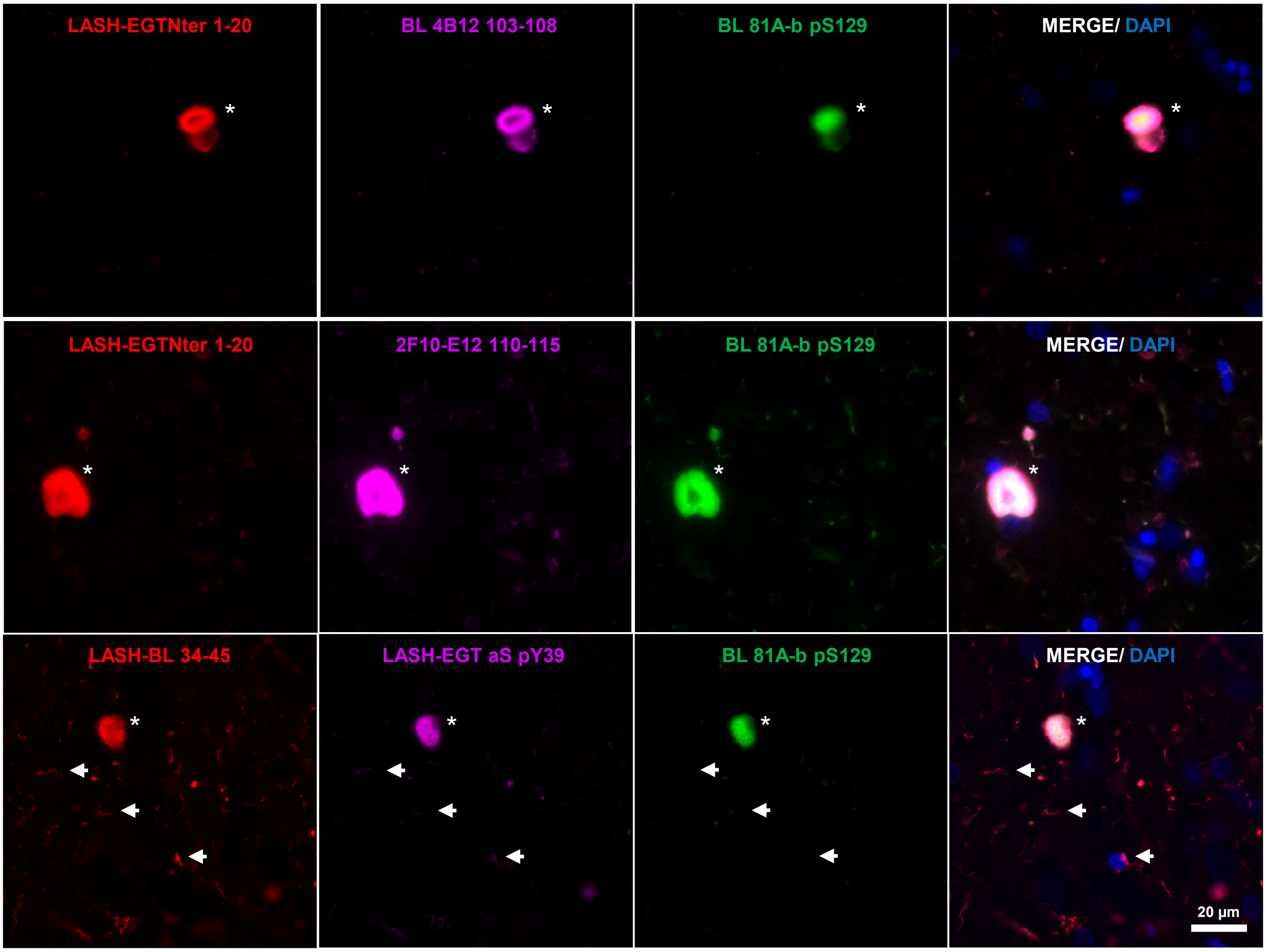

Supplementary Figure 7: IF labelling of PD cingulate cortex using the aSyn N-terminal LASH-EGTNter 1-20 or LASH-BL 34-45, aSyn C-terminal BL 4B12 103-108 or AB 134-138, and aSyn pS129 BL 81A-biotin antibodies. LBs are marked with asterisks, and LNs with arrows. Representative images from PD1 cingulate cortex are taken using Leica DM5500 B upright microscope at 20x magnification. aSyn = alpha-synuclein; DAPI = 4',6-diamidino-2-phenylindole; IF = immunofluorescence; IHC = immunohistochemistry; LB = Lewy body; LN = Lewy neurite; PD = Parkinson’s disease

SUPPLEMENTARY FIGURE 8

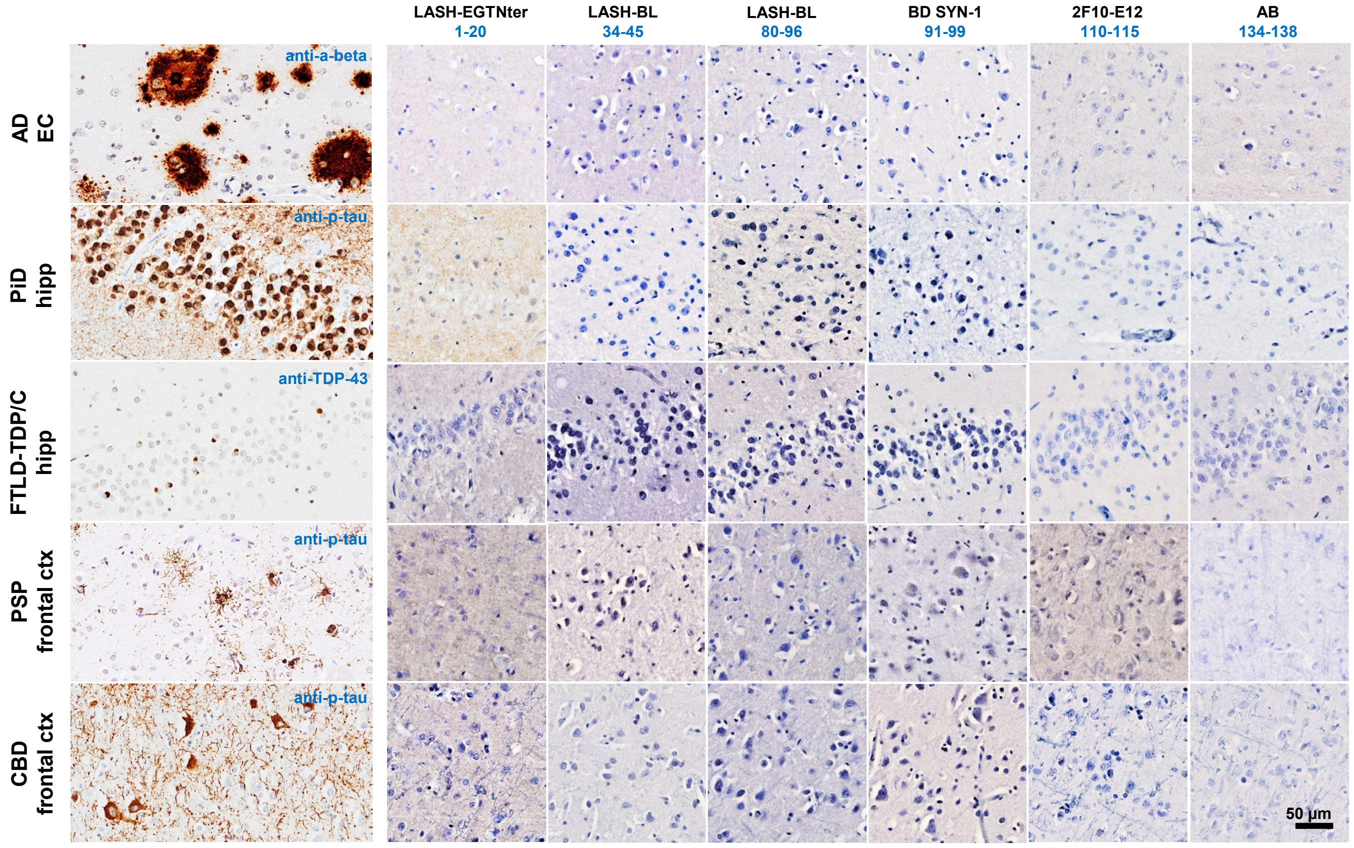

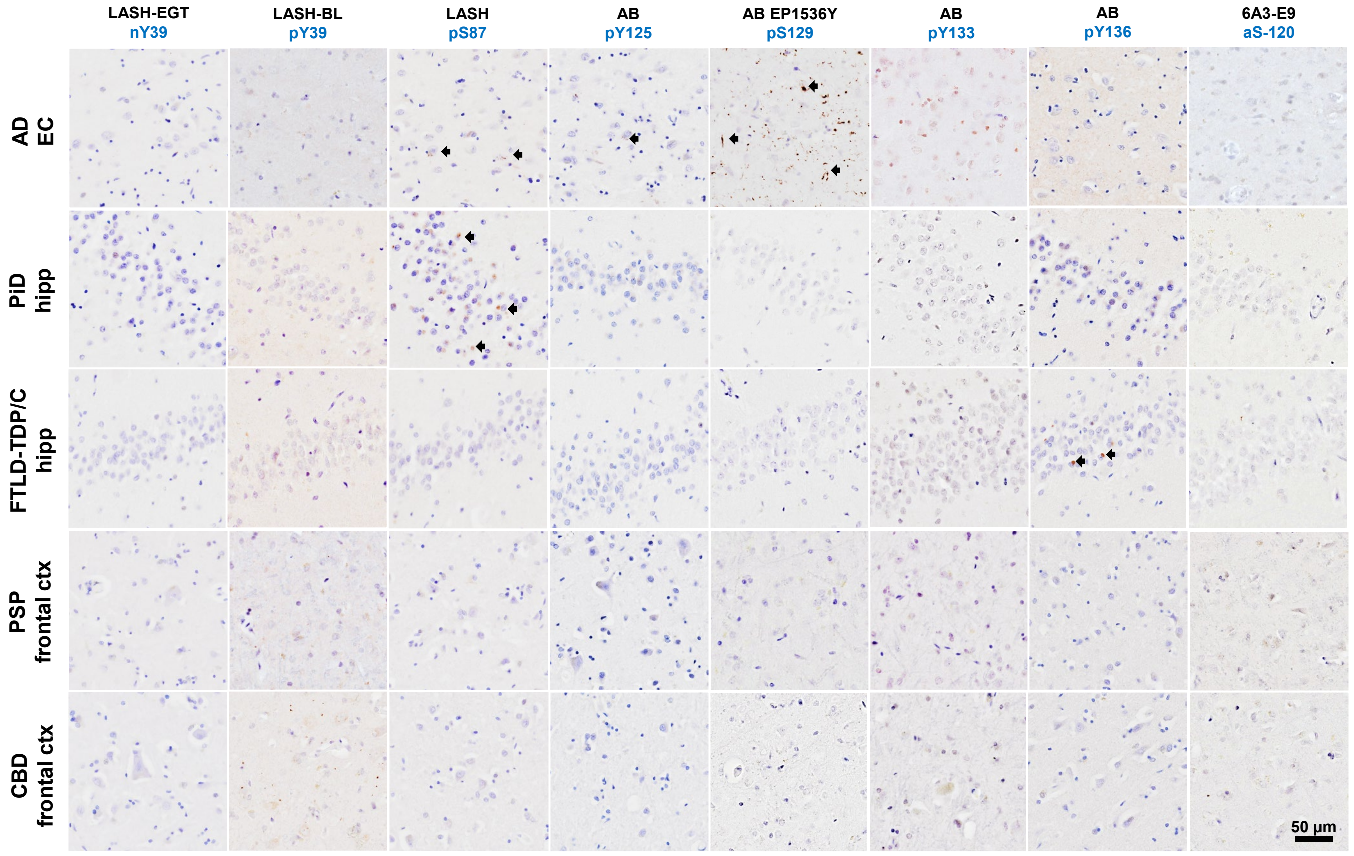

Supplementary Figure 8: Specificity validation of aSyn antibodies on human post-mortem tissues via IHC. The frontal cortices of PSP and CBD, and the hippocampi and the EC of AD, PiD and FTLD-TDP/C were stained using the selection of aSyn non-modified and PTM antibodies. Cross-reactivity was not observed with pathological accumulations positive for a-beta (antibody: Agilent anti-a-beta), p-tau (antibody: TF AT8) or TDP-43 (antibody: LSBio 6F/3D). Arrows indicate aSyn-positive structures detected on each tissue. Representative images taken from the entorhinal cortices (layers V-VI) and hippocampi (dentate gyrus) of AD1-2, PiD1-2 and FTLP-TDP/C1-2, and the frontal cortices (layers V–VI) of PSP1-2 and CBD1-2. a-beta = amyloid-beta; AD = Alzheimer's disease; aSyn = alpha-synuclein; CBD = corticobasal degeneration; ctx = cortex; EC = entorhinal cortex; FTLD-TDP/C = frontotemporal lobar degeneration of transactive response DNA-binding protein 43 type C; hipp = hippocampus; IHC = immunohistochemistry; PiD = Pick's disease; PSP = posterior supranuclear palsy; p-tau = phosphorylated tubulin-associated unit; PTM = post-translational modification; TDP-43 = transactive response DNA binding protein 43kDa

SUPPLEMENTARY FIGURE 9

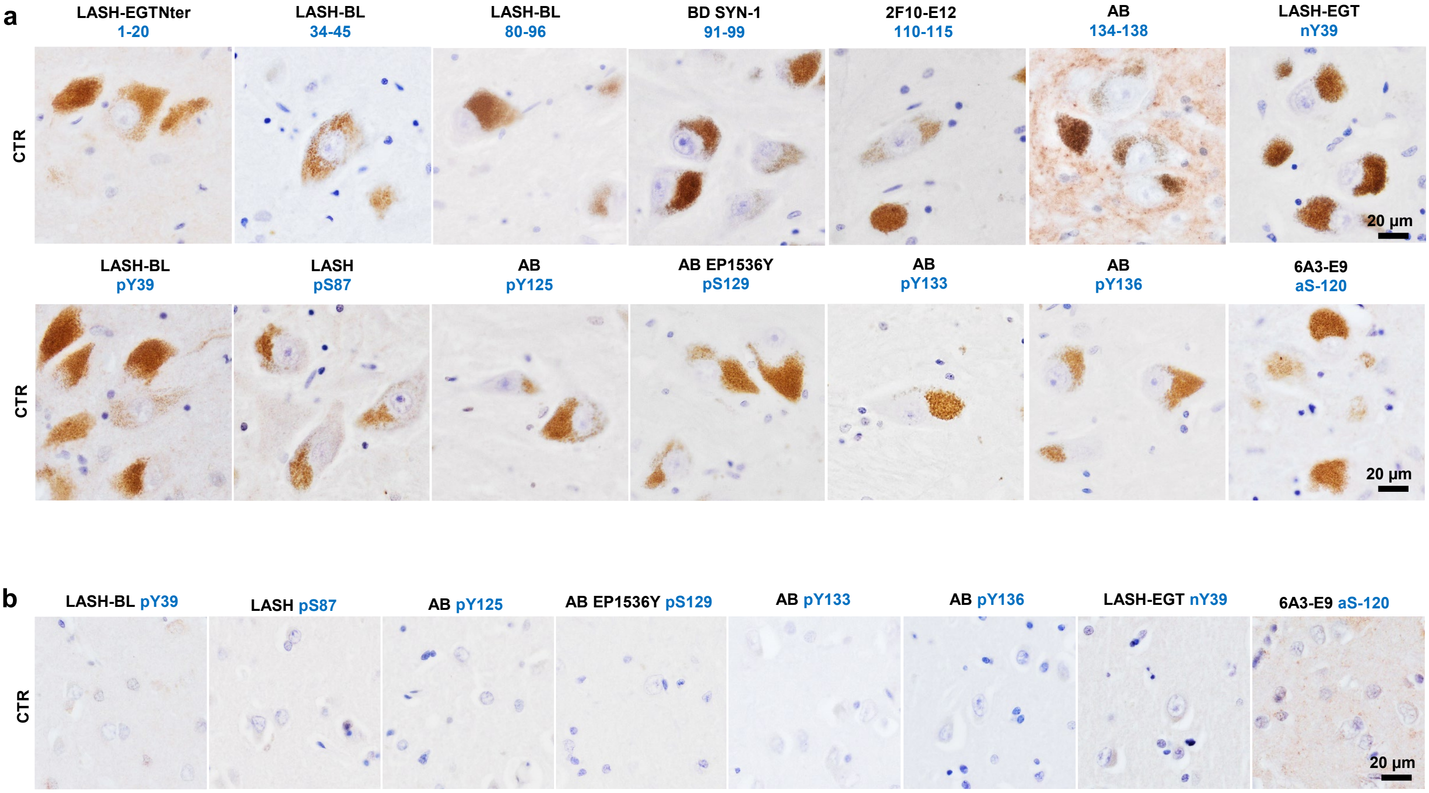

Supplementary Figure 9: Complementary images to Figure 5b-c. Staining of the **(a)** SN and **(b)** cingulate cortex (layers V–VI) of healthy controls with aSyn antibodies. aSyn = alpha-synuclein; CTR = control; SN = substantia nigra

SUPPLEMENTARY FIGURE 10

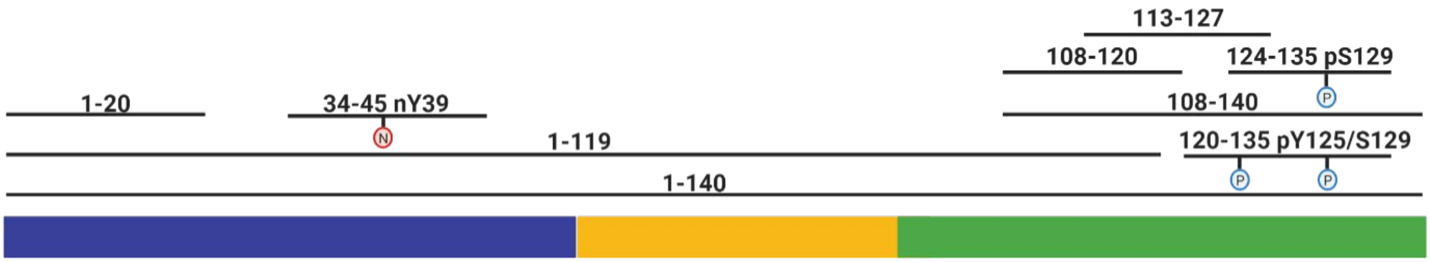

Supplementary Figure 10: A schematic of the aSyn human proteins and peptides used for BALB/c mouse immunization. Schematic created with Biorender.com (agreement no: NS23V1NZV8).

Western blots uncropped

FIGURE 3

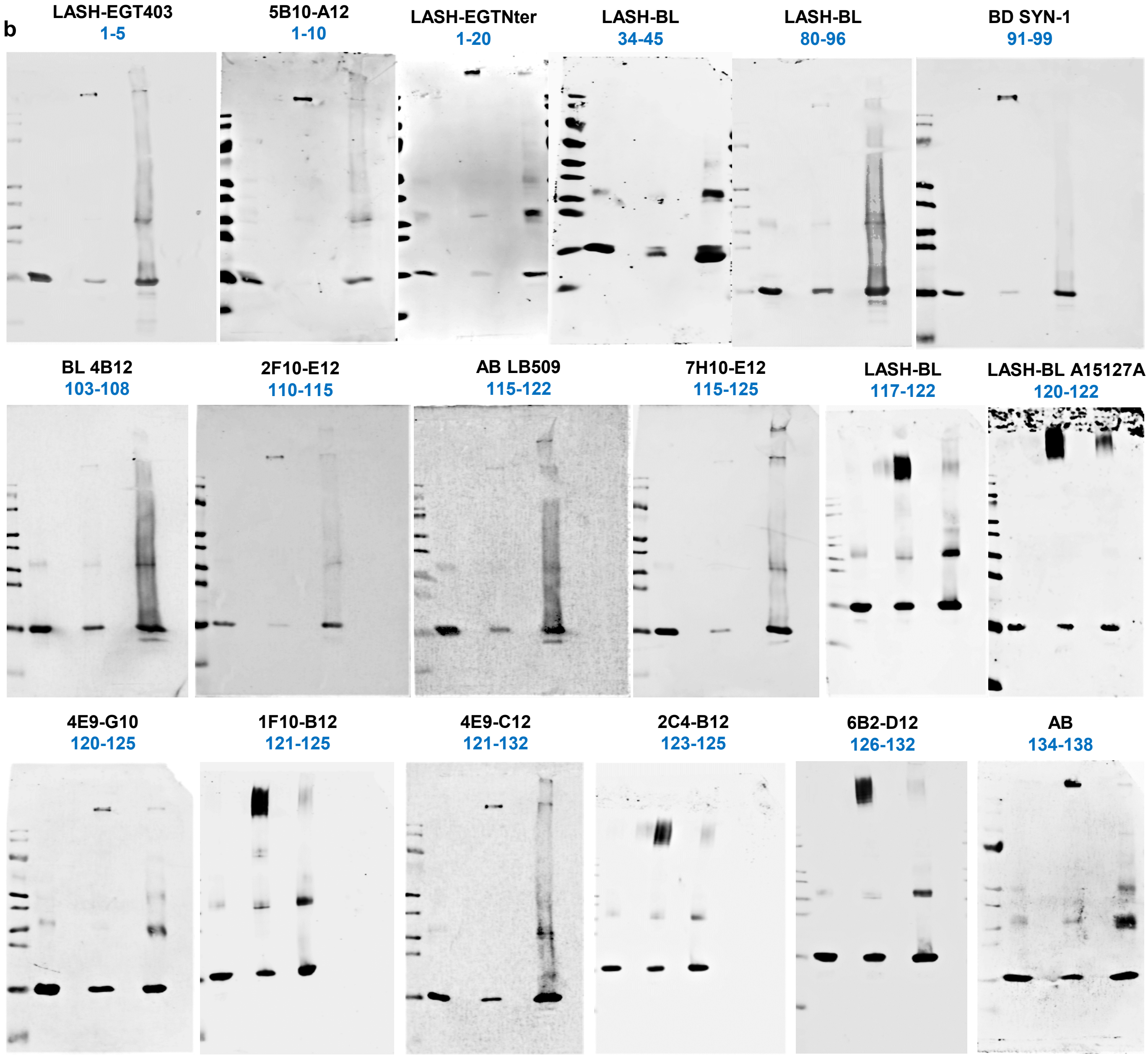

Western blots uncropped

SUPPLEMENTARY FIGURE 1

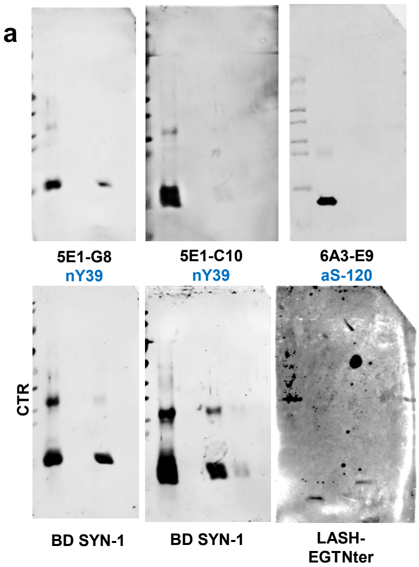

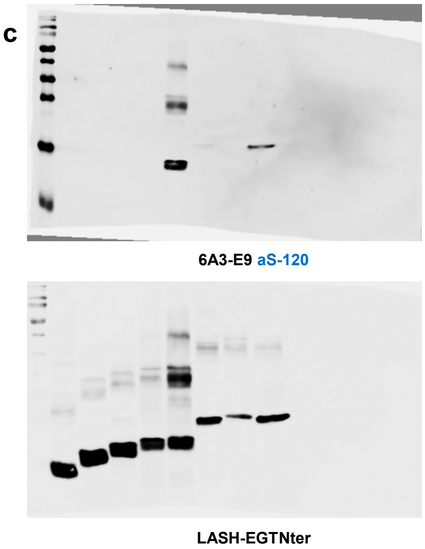

Western blots uncropped

SUPPLEMENTARY FIGURE 5

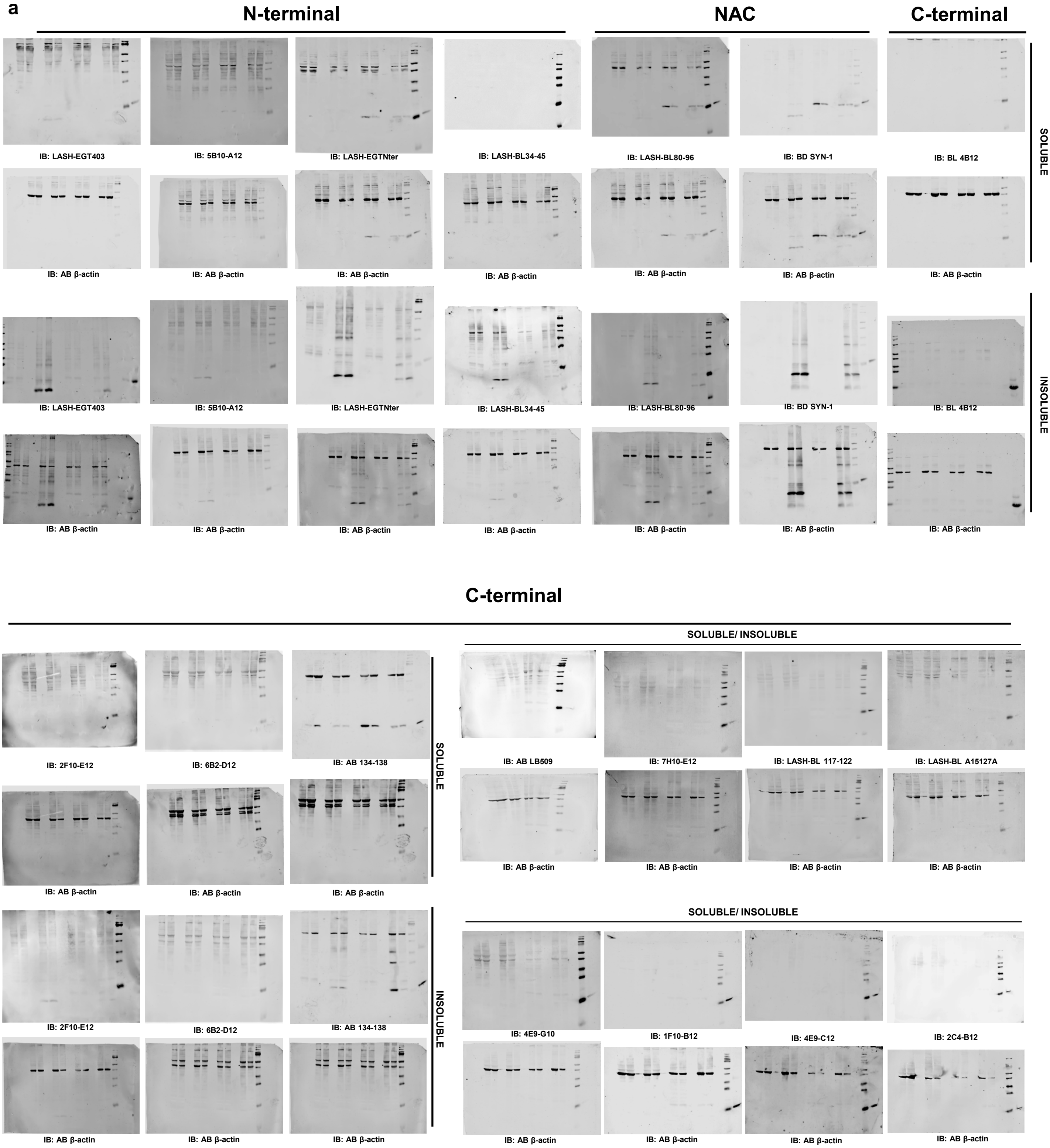

Western blots uncropped

SUPPLEMENTARY FIGURE 5

b

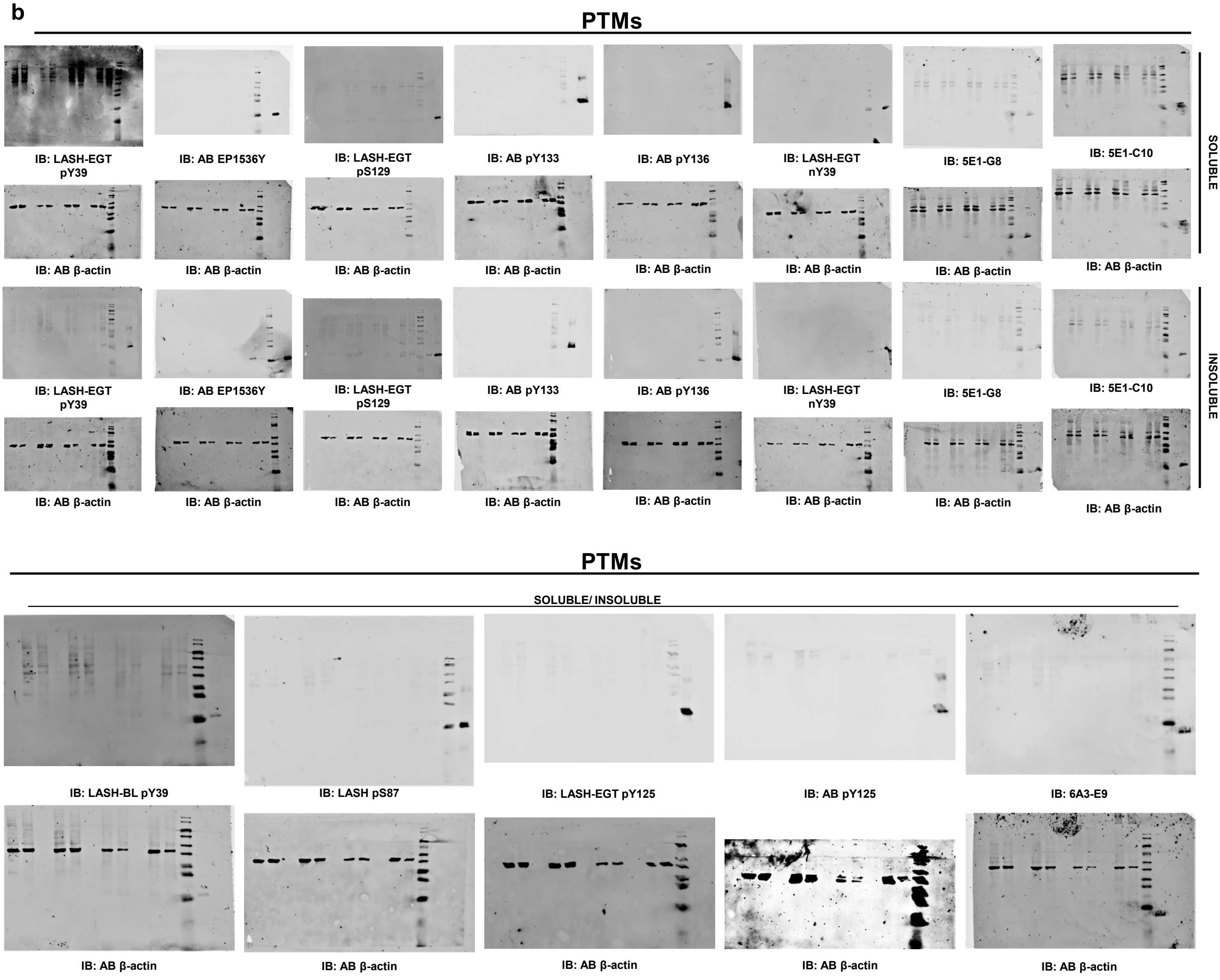

SUPPLEMENTARY TABLES

Supplementary Table 1: A list of all the programmes initialised to generate monoclonal antibodies against aSyn.

programme	immunogen	carrier	antigen sequence
LASH-EGT403	UniProtKB - P37840 (SYUA_HUMAN) **1-20**	KLH	MDVFMKGLSK AKEGVVAAAE
LASH-EGT404	UniProtKB - P37840 (SYUA_HUMAN) **1-140**	na	recombinant aSyn human FL (1-140)
LASH-EGT405	UniProtKB - P37840 (SYUA_HUMAN) **113-127**	KLH	LEDMPVDP DNEAYEM
LASH-EGT406	UniProtKB - P37840 (SYUA_HUMAN) **108-120**	KLH	PQE GILEDMPVDP
LASH-EGT407	UniProtKB - P37840 (SYUA_HUMAN) **124-135 pS129**	KLH	AYEMP(pS)E EGYQD
LASH-EGT408	UniProtKB - P37840 (SYUA_HUMAN) **108-140**	na	PQE GILEDMPVDP DNEAYEMPSE EGYQDYEPEA
LASH-EGT409	UniProtKB - P37840 (SYUA_HUMAN) **120-135 pY125/pS129**	KLH	P DNEA(pY)EM P(pS)E EGYQD
LASH-EGT410	UniProtKB - P37840 (SYUA_HUMAN) **1-119**	na	recombinant aSyn human 1-119
LASH-EGT416	UniProtKB - P37840 (SYUA_HUMAN) **34-45 nY39**	KLH	KEGVL(nY)VGSKTK

aSyn = alpha-synuclein; FL = full-length; KLH = keyhole limpet hemocyanin; na = not available

Supplementary Table 2: A list of all aSyn proteins and peptides included in this study.

aSyn protein/ peptide	Mw/ Da	aSyn protein/ peptide	Mw/ Da	aSyn protein/ peptide	Mw/ Da
h/m 1-11	1,227	h 39-140	10,600	h pS87	14,540
h 1-101	10,062	h 65-140	7,872	h pY125	14,540
h 1-110	11,045	h 71-140	7,374	h pY125/S129	14,620
h 1-114	11,457	h Δ111-115	13,933	h pS129	14,540
h 1-115	11,572	h Δ111-115/133-135	13,526	h pY125/pY133/pY136	14,745
h 1-119	12,015	h Δ120-125	13,770	m 1-120	12,222
h 1-120	12,112	h Δ133-135	14,054	m Δ120-125	13,881
h 1-122	12,341	h FL	14,460	m Δ133-135	14,079
h 1-123	12,470	h Y39F	14,444	m 20-140	12,551
h 1-124	12,541	h Y133/136F nY39	14,490	m FL	14,485
h 1-133	13,627	h nY39	14,522	m E114A	14,427
h 1-135	13,871	h nY125	14,522	m D115A	14,441
h 5-140	13,968	h pY39	14,540	m pS129	14,580

aSyn = alpha-synuclein; Da = dalton; FL = full-length; h = human; m = mouse; Mw = molecular weight

Supplementary Table 3: A list of all the purified antibodies and their epitopes after finalisation of the monoclonal antibody generation programmes.

antibody full name	antibody short name	epitope	species/ clonality	isotype	reactivity	concentration (mg/mL)	amount (mg)
LASH-EGT403 6D10-F10	LASH-EGT403	1-5	mus mc	IgG1 K	hu, mus	1.1	56.2
LASH-EGT410 5B10-A12	5B10-A12	1-10	mus mc	IgG1 K	hu, mus	1.4	56.2
LASH-EGT406 2F10-E12	2F10-E12	110-115	mus mc	IgG1 K	hu, mus	1.8	79.7
LASH-EGT405 7H10-E12	7H10-E12	115-125	mus mc	IgG1 K	hu	1.6	38.4
LASH-EGT408 4E9-C12	4E9-C12	121-132	mus mc	IgG1 K	hu	1.2	35.5
LASH-EGT405 2C4-B12	2C4-B12	123-125	mus mc	IgG1 K	hu	1.0	28.7
LASH-EGT408 4E9-G10	4E9-G10	120-125	mus mc	IgG1 K	hu	0.9	26.5
LASH-EGT410 6B2-D12	6B2-D12	126-132	mus mc	IgG1 K	hu, mus	1.2	37.2
LASH-EGT410 1F10-B12	1F10-B12	121-125	mus mc	IgG1 K	hu	1.2	36.7
LASH-EGT406 6A3-E9	6A3-E9	aSyn-120	mus mc	IgG1 K	hu	1.0	23.0
LASH-EGT416 5E1-G8	5E1-G8	nY39	mus mc	IgG1 K	hu, mus	1.3	52.2
LASH-EGT416 5E1-C10	5E1-C10	nY39	mus mc	IgG1 K	hu, mus	1.3	53.1

aSyn = alpha-synuclein; hu = human; IgG = immunoglobulin G; K = kappa; mc = monoclonal; mus = mouse

Supplementary Table 4: A list of all the primary and secondary antibodies used in this study.

primary antibodies						
**antibody name**	**epitope**	**species/ clonality**	**reactivity**	**concentration (mg/mL)**	**company**	**catalogue #**
LASH-EGT403	aSyn 1-5	mus mc	h, m	1.12	-	-
5B10-A12	aSyn 1-10	mus mc	h, m	1.40	-	-
LASH-EGTNter	aSyn 1-20	rab pc	h, m	1.34	-	-
LASH-BL 34-45	aSyn 34-45	mus mc	h, m	1.00	Biolegend	849102
LASH-BL 80-96	aSyn 80-96	mus mc	h, m	1.00	Biolegend	848302
BD SYN-1	aSyn 91-99	mus mc	h, m	0.25	BD Transduction	BD610787
BL 4B12	aSyn 103-108	mus mc	h	1.00	Biolegend	807801
2F10-E12	aSyn 110-115	mus mc	h, m	1.81	-	-
AB LB509	aSyn 115-122	mus mc	h	1.00	Abcam	ab27766
7H10-E12	aSyn 115-125	mus mc	h	1.60	-	-
LASH-BL 117-122	aSyn 117-122	mus mc	h	1.53	Biolegend	848601
LASH-BL A15127A	aSyn 120-122	mus mc	h	5.19	Biolegend	848401
4E9-G10	aSyn 120-125	mus mc	h	0.89	-	-
1F10-B12	aSyn 121-125	mus mc	h	1.23	-	-
4E9-C12	aSyn 121-132	mus mc	h	1.18	-	-
2C4-B12	aSyn 123-125	mus mc	h	0.96	-	-
6B2-D12	aSyn 126-132	mus mc	h, m	1.24	-	-
AB 134-138	aSyn 134-138	rab pc	h, m	1.00	Abcam	ab131508
LASH-BL pY39	aSyn pY39	mus mc	h	1.00	Biolegend	849201
LASH-EGT pY39	aSyn pY39	rab pc	h, m	0.50	-	-
LASH pS87	aSyn pS87	rab pc	h	0.40	-	-
LASH-EGT pY125	aSyn pY125	rab pc	h	0.20	-	-
AB pY125	aSyn pY125	rab pc	h	0.50	Abcam	ab10789
AB EP1536Y	aSyn pS129	rab mc	h, m	2.68	Abcam	ab51253
LASH-EGT pS129	aSyn pS129	rab pc	h, m	0.10	-	-
BL 81A	aSyn pS129	mus mc	h, m	1.00	Biolegend	825701
BL 81A biotin	aSyn pS129	mus mc	h, m	0.50	Biolegend	824704
AB MJF-R13	aSyn pS129	rab mc	h, m	4.20	Abcam	ab168381
AB pY133	aSyn pY133	rab pc	h, m	1.50	Abcam	ab194910
AB pY136	aSyn pY136	rab pc	h, m	1.00	Abcam	ab131491
LASH-EGT nY39	aSyn nY39	rab pc	h, m	0.53	-	-
5E1-G8	aSyn nY39	mus mc	h, m	1.31	-	-
5E1-C10	aSyn nY39	mus mc	h, m	1.27	-	-
6A3-E9	aSyn-120	mus mc	h	0.96	-	-
AB β-actin AC-15	β-actin	mus mc	h, m	1.00	Abcam	ab6276
AB anti-MAP2	MAP2	ch pc	m, r	na	Abcam	ab5392
TF AT8	tau pS202/T205	mus mc	h, m, r	0.20	ThermoFisher	MN1020
Agilent 6F/3D	a-beta	mus mc	h	na	Agilent	M0872
LSBio 2E2-D3	TDP-43	mus mc	h	0.48	LSBio	LS-B4521

secondary antibodies			
**antibody name**	**concentration (mg/mL)**	**company**	**catalogue #**
DAPI 461	2.00	ThermoFisher	D1306
goat anti-mouse Alexa Fluor 488	2.00	ThermoFisher	A-11029
donkey anti-rabbit Alexa Fluor 488	2.00	ThermoFisher	A-21206
goat anti-chicken Alexa Fluor 568	2.00	ThermoFisher	A-11041
donkey anti-rabbit Alexa Fluor 568	2.00	ThermoFisher	A-10042
donkey anti-mouse Alexa Fluor 647	2.00	ThermoFisher	A-31571
donkey anti-rabbit Alexa Fluor 647	2.00	ThermoFisher	A-31573
IRDye 680RD goat anti-mouse	-	Li-Cor	926-68070
IRDye 800CW goat anti-rabbit	-	Li-Cor	926-32211

a-beta = amyloid-beta; aSyn = alpha-synuclein; ch = chicken; h = human; MAP2 = microtubule-associated protein 2; mc = monoclonal; mus or m = mouse; pc = polyclonal; rab or r = rabbit; tau = tubulin-associated unit; TDP-43 = transactive response DNA-binding protein 43kDa

Supplementary Table 5: Optimised IHC settings for the antibodies used on human post-mortem brain tissues.

antibody	epitope	IHC: dilution	IHC: antigen retrieval	IF: dilution*
LASH-EGT403	aSyn 1-5	1:50	FA	na
5B10-A12	aSyn 1-10	1:5,000	AC+FA	na
LASH-EGTNter	aSyn 1-20	1:15,000	AC+FA	1:2,000
LASH-BL 34-45	aSyn 34-45	1:30,000	FA	1:10,000
LASH-BL 80-96	aSyn 80-96	1:20,000	FA	na
BD SYN-1	aSyn 91-99	1:5,000	FA	na
BL 4B12	aSyn 103-108	1:100,000	AC+FA	1:5,000
2F10-E12	aSyn 110-115	1:10,000	FA	1:500
AB LB509	aSyn 115-122	1:25,000	AC+FA	na
7H10-E12	aSyn 115-125	1:9,000	AC+FA	na
LASH-BL 117-122	aSyn 117-122	1:80,000	AC+FA	na
LASH-BL A15127A	aSyn 120-122	1:15,000	AC+FA	na
4E9-G10	aSyn 120-125	1:4,000	AC+FA	na
1F10-B12	aSyn 121-125	1:15,000	AC+FA	na
4E9-C12	aSyn 121-132	1:10,000	AC+FA	na
2C4-B12	aSyn 123-125	1:12,000	AC+FA	na
6B2-D12	aSyn 126-132	1:500	FA	na
AB 134-138	aSyn 134-138	1:25,000	AC+FA	1:100
LASH-BL pY39	aSyn pY39	1:2,000	AC+FA	na
LASH-EGT pY39	aSyn pY39	1:500	AC+FA	1:50
LASH pS87	aSyn pS87	1:600	FA	na
LASH-EGT pY125	aSyn pY125	1:100	FA	na
AB pY125	aSyn pY125	1:500	FA	na
AB EP1536Y	aSyn pS129	1:60,000	AC+FA	na
LASH-EGT pS129	aSyn pS129	1:200	FA	na
AB pY133	aSyn pY133	1:400	AC+FA	na
AB pY136	aSyn pY136	1:100	FA	na
LASH-EGT nY39	aSyn nY39	1:1,000	FA	na
5E1-G8	aSyn nY39	1:4,000	AC+FA	na
5E1-C10	aSyn nY39	1:250	AC+FA	na
6A3-E9	aSyn-120	1:2,500	AC+FA	na
BL 81A biotin	aSyn pS129	na	na	1:500
TF AT8	tau pS202/T205	1:600	AC+FA	na
Agilent 6F/3D	a-beta	1:100	AC+FA	na
LSBio 2E2-D3	TDP-43	1:8,000	AC	na

*AC+FA pre-treatment was applied in IF studies for all antibodies. a-beta = amyloid-beta; AC = autoclave; aSyn = alpha-synuclein; FA = formic acid; IF = immunofluorescence; IHC = immunohistochemistry; na = not applicable; tau = tubulin associated unit; TDP-43 = transactive response DNA-binding protein 43kDa
