# Supplementary material for: Development and validation of an expanded antibody toolset that captures alpha-synuclein pathological diversity in Lewy body diseases
Source: NPJ Parkinsons Dis. 2023 Dec 7;9:161. doi: 10.1038/s41531-023-00604-y (PMC10703845; doi:10.1038/s41531-023-00604-y)
Supplement: Supplementary file 2 — Reporting Summary [file 41531_2023_604_MOESM2_ESM.pdf]

nature portfolio

Corresponding author(s): Hilal A. Lashuel

Last updated by author(s): Oct 18, 2023

Reporting Summary

Nature Portfolio wishes to improve the reproducibility of the work that we publish. This form provides structure for consistency and transparency in reporting. For further information on Nature Portfolio policies, see our Editorial Policies and the Editorial Policy Checklist.

Statistics

For all statistical analyses, confirm that the following items are present in the figure legend, table legend, main text, or Methods section.

n/a	Confirmed	
☒	☐	The exact sample size (*n*) for each experimental group/condition, given as a discrete number and unit of measurement
☒	☐	A statement on whether measurements were taken from distinct samples or whether the same sample was measured repeatedly
☒	☐	The statistical test(s) used AND whether they are one- or two-sided<br>*Only common tests should be described solely by name; describe more complex techniques in the Methods section.*
☒	☐	A description of all covariates tested
☒	☐	A description of any assumptions or corrections, such as tests of normality and adjustment for multiple comparisons
☒	☐	A full description of the statistical parameters including central tendency (e.g. means) or other basic estimates (e.g. regression coefficient) AND variation (e.g. standard deviation) or associated estimates of uncertainty (e.g. confidence intervals)
☒	☐	For null hypothesis testing, the test statistic (e.g. *F*, *t*, *r*) with confidence intervals, effect sizes, degrees of freedom and *P* value noted<br>*Give P values as exact values whenever suitable.*
☒	☐	For Bayesian analysis, information on the choice of priors and Markov chain Monte Carlo settings
☒	☐	For hierarchical and complex designs, identification of the appropriate level for tests and full reporting of outcomes
☒	☐	Estimates of effect sizes (e.g. Cohen's *d*, Pearson's *r*), indicating how they were calculated

Our web collection on statistics for biologists contains articles on many of the points above.

Software and code

Policy information about availability of computer code

Data collection	Akta 25 FPLC system (RRID:SCR_023461), Biacore 8K (GE Healthcare #29215379), Li-Cor Odyssey CLx imaging system (RRID:SCR_014579), Tecan Infinite M200 microplate reader (RRID:SCR_019033), Zeiss LSM 700 (RRID:SCR_017377), Leica DM5500 B upright microscope (RRID:SCR_020219), Olympus VS120 microscope (RRID:SCR_018411)
Data analysis	Image Studio Lite5.2 (RRID:SCR_013715), Zen Digital Imaging software (RRID: SCR_013672), Leica Application Suite X (RRID:SCR_013673), Olympus VS120 microscope (RRID:SCR_018411), QuPath (RRID:SCR_018257)

For manuscripts utilizing custom algorithms or software that are central to the research but not yet described in published literature, software must be made available to editors and reviewers. We strongly encourage code deposition in a community repository (e.g. GitHub). See the Nature Portfolio guidelines for submitting code & software for further information.

Data

Policy information about availability of data

All manuscripts must include a data availability statement. This statement should provide the following information, where applicable:

- Accession codes, unique identifiers, or web links for publicly available datasets
- A description of any restrictions on data availability
- For clinical datasets or third party data, please ensure that the statement adheres to our policy

Provide your data availability statement here.

Research involving human participants, their data, or biological material

Policy information about studies with human participants or human data. See also policy information about sex, gender (identity/presentation), and sexual orientation and race, ethnicity and racism.

Reporting on sex and gender	The sex (biological attribute) of the post-mortem brain sample donors were indicated in the manuscript, in accordance with the SAGER guidelines.
Reporting on race, ethnicity, or other socially relevant groupings	na
Population characteristics	na
Recruitment	na
Ethics oversight	na

Note that full information on the approval of the study protocol must also be provided in the manuscript.

Field-specific reporting

Please select the one below that is the best fit for your research. If you are not sure, read the appropriate sections before making your selection.

☒ Life sciences ☐ Behavioural & social sciences ☐ Ecological, evolutionary & environmental sciences

For a reference copy of the document with all sections, see nature.com/documents/nr-reporting-summary-flat.pdf

Life sciences study design

All studies must disclose on these points even when the disclosure is negative.

Sample size	Sample size was selected based on sample availability and to comply with the minimum sample size requirements.
Data exclusions	No data were excluded.
Replication	IHC on post-mortem tissues were run on three independent laboratories and attempts at replication were successful.
Randomization	No experiments were conducted requiring randomization.
Blinding	No experiments were conducted requiring blinding.

Reporting for specific materials, systems and methods

We require information from authors about some types of materials, experimental systems and methods used in many studies. Here, indicate whether each material, system or method listed is relevant to your study. If you are not sure if a list item applies to your research, read the appropriate section before selecting a response.

Materials & experimental systems

n/a	Involved in the study
☐	☒ Antibodies
☐	☒ Eukaryotic cell lines
☒	☐ Palaeontology and archaeology
☐	☒ Animals and other organisms
☒	☐ Clinical data
☒	☐ Dual use research of concern
☒	☐ Plants

Methods

n/a	Involved in the study
☒	☐ ChIP-seq
☒	☐ Flow cytometry
☒	☐ MRI-based neuroimaging

Antibodies

Antibodies used	in-house antibodies: LASH-EGT403, 5B10-A12, LASH-EGTNter, 2F10-E12, 7H10-E12, 4E9-G10, 1F10-B12, 4E9-C12, 2C4-B12, 6B2-D12, LASH-EGT pY39, LASH pS87, LASH-EGT pY125, LASH-EGT pS129, LASH-EGT nY39, 5E1-G8, 5E1-C10, 6A3-E9; commercial antibodies: LASH-BL 34-45 (Biolegen #849102), LASH-BL 80-96 (Biolegend #848302), BD SYN-1 (BD Transduction #BD610787), BL 4B12

	(Biolegend #807801), AB LB509 (Abcam #ab27766), LASH-BL 117-122 (Biolegend #848601), LASH-BL A15127A (Biolegend #848401), AB 134-138 (Abcam #ab131508), LASH-BL pY39 (Biolegend #849201), AB pY125 (Abcam #ab10789), AB EP1536Y (Abcam #ab51253), BL 81A (Biolegend #825701), BL 81A biotin (Biolegend #824704), AB MJF-R13 (Abcam #ab168381), AB pY133 (Abcam #ab194910), AB pY136 (Abcam #ab131491), AB β-actin AC-15 (Abcam #ab6276), AB anti-MAP2 (Abcam #ab5392), TF AT8 (ThermoFisher #MN1020), Agilent 6F/3D (Agilent #M0872), LSBio 2E2-D3 (LSBio #LS?B4521), DAPI 461 (ThermoFisher #D1306), goat anti-mouse Alexa Fluor 488 (ThermoFisher #A-11029), donkey anti-rabbit Alexa Fluor 488 (ThermoFisher #A-21206), goat anti-chicken Alexa Fluor 568 (ThermoFisher #A-11041), donkey anti-rabbit Alexa Fluor 568 (ThermoFisher #A-10042), donkey anti-mouse Alexa Fluor 647 (ThermoFisher #A-31571), donkey anti-rabbit Alexa Fluor 647 (ThermoFisher #A-31573), IRDye 680RD goat anti-mouse (Li-Cor #926-68070), IRDye 800CW goat anti-rabbit (Li-Cor #926-32211)
Validation	all the validation experiments are detailed in the manuscript

Eukaryotic cell lines

Policy information about cell lines and Sex and Gender in Research

Cell line source(s)	C57BL/6JRj male mice (Janvier Labs) and aSyn KO male mice (C57BL/6J OlaHsd, Harlan)
Authentication	All animal experimentation was performed in compliance with the European Communities Council Directive of 24 November 1986 (86/609EEC) and with approval of the Cantonal Veterinary Authorities (Vaud, Switzerland) and the Swiss Federal Veterinary Office (authorization number VD2067.2).
Mycoplasma contamination	Primary neurons were not tested for mycoplasma contamination.
Commonly misidentified lines (See ICLAC register)	na

Animals and other research organisms

Policy information about studies involving animals; ARRIVE guidelines recommended for reporting animal research, and Sex and Gender in Research

Laboratory animals	C57BL/6JRj male mice (Janvier Labs) and aSyn KO male mice (C57BL/6J OlaHsd, Harlan) - fibril injection at 3mo and sacrifice at 6mo of age
Wild animals	na
Reporting on sex	The primary neurons were extracted from both male and female pups and results apply to both sexes; the IHC on tissues were run on brains of male mice only (wild-type and aSyn knockout) - these brains were left over after behavioural experiments for another project, and our approach involved a reduction of the experimental animals used, in line with 3R principles.
Field-collected samples	na
Ethics oversight	All animal experimentation was performed in compliance with the European Communities Council Directive of 24 November 1986 (86/609EEC) and with approval of the Cantonal Veterinary Authorities (Vaud, Switzerland) and the Swiss Federal Veterinary Office (authorization number VD2067.2).

Note that full information on the approval of the study protocol must also be provided in the manuscript.
